# Effect of miR-21 in mesenchymal stem cells-derived extracellular vesicles behavior

**DOI:** 10.1186/s13287-023-03613-z

**Published:** 2023-12-21

**Authors:** Miriam Morente-López, Rocio Mato-Basalo, Sergio Lucio-Gallego, Concha Gil, Mónica Carrera, Juan A. Fafián-Labora, Jesús Mateos, María C. Arufe

**Affiliations:** 1 Grupo de Terapia Celular y Medicina Regenerativa, Dpto. de Fisioterapia, Medicina y Ciencias Biomédicas. Facultad de Ciencias de la Salud, Universidade da Coruña, INIBIC-CHUAC, CICA, 15006 A Coruña, Spain; 2 Proteomics Facility-Complutense University and Scientific Park Foundation of Madrid, Madrid, Spain; 3 Institute of Marine Research (IIM), Spanish National Research Council (CSIC), Vigo, Spain; 4 Clinical Pharmacology Group, Health Research Institute of Santiago de Compostela (FIDIS), 15706 Santiago de Compostela, Spain

**Keywords:** Mesenchymal stem cells (MSC), Extracellular vesicles (EV), miR-21-5p (miR-21), Syndecan-1 (SDC1), Senescence-associated secretory phenotype (SASP), Inflammaging

## Abstract

**Background:**

A challenging new branch of research related to aging-associated diseases is the identification of miRNAs capable of modulating the senescence-associated secretory phenotype (SASP) which characterizes senescent cells and contributes to driving inflammation.

**Methods:**

Mesenchymal stem cells (MSC) from human umbilical cord stroma were stable modified using lentivirus transduction to inhibit miR-21-5p and shotgun proteomic analysis was performed in the MSC-derived extracellular vesicles (EV) to check the effect of miR-21 inhibition in their protein cargo. Besides, we studied the paracrine effect of those modified extracellular vesicles and also their effect on SASP.

**Results:**

Syndecan-1 (SDC1) was the most decreased protein in MSC-miR21^−^-derived EV, and it was involved in inflammation and EV production. MSC-miR21^−^-derived EV were found to produce a statistically significant inhibitory effect on SASP and inflammaging markers expression in receptor cells, and in the opposite way, these receptor cells increased their SASP and inflammaging expression statistically significantly when treated with MSC-miR-21^+^-derived EV.

**Conclusion:**

This work demonstrates the importance of miR-21 in inflammaging and its role in SASP through SDC1.

**Graphical abstract:**

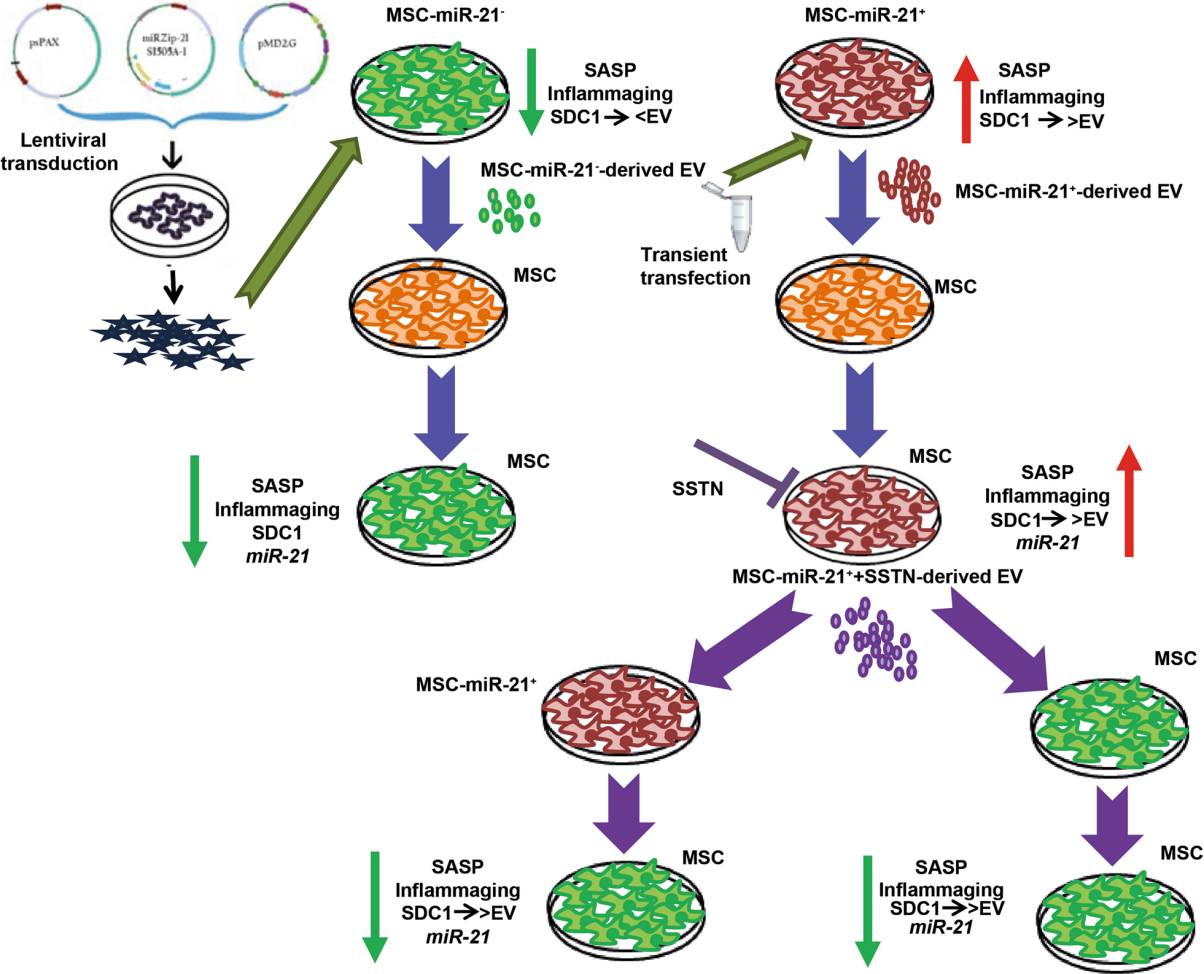

**Supplementary Information:**

The online version contains supplementary material available at 10.1186/s13287-023-03613-z.

## Introduction

Exosomes and small extracellular vesicles are included in extracellular vesicles (EV) produced by cells; they contain a lot of bioactive molecules such as miRNA and proteins. EV are acting as a vehicle for cell–cell communication to modulate cellular activities and influencing the expression of their target genes in the receptor cells [28]. In recent years, MSC-derived EV are replacing MSCs themselves due to their paracrine functions, known as the new cell-free alternatives to MSC-based cell therapy. These MSC-derived EV show advantages over self-MSC including greater stability, lower immunogenicity and convenience for transportation, storage and administration compared to cellular therapy. Constituting one of the most promising lines of research in regenerative medicine due to its therapeutic potential [8].

The senescence-associated secretory phenotype (SASP) is being seen to offer a new perspective as a marker for the progression of many age-related diseases. Being able to give us an idea of the state of inflammation and complexity of multiple diseases associated with age from cancer to neurodegeneration [5]. Numerous miRNAs influence senescence by modulating the abundance of key senescence-regulating proteins, generally by reducing the stability and/or translation of the mRNAs encoding these factors. Consequently, miRNAs influence SASP and their manipulation will allow diagnostic and therapeutic opportunities targeting senescent cells [19]. There are many expectations about the therapeutic use of miRNAs transported by MSC-derived EV in autoimmune diseases. Previous investigations carried out in the group consisted of EV treatment of MSC previously modified to contain a miR-21 antagonist. These EVs were found to be more effective in reducing systemic inflammation in age-related diseases such as osteoarthritis (OA) than miR-21-inhibited MSC themselves. In addition, their MSC-miR-21-derived EV modulated the ERK1/2 family to exert its anti-inflammatory effect in the studied in vivo OA model [18].

In the present work, a *shotgun* proteomic analysis was performed using TMT (10 plex) to compare MSC-miR-21^−^-derived EV in front of MSC-derived EV and discover the modulated proteins associated with inflammaging. The target found out, Syndecan-1 (SDC1), is a cell surface heparan sulfate proteoglycan, associates directly with the αvβ3 and αvβ5 integrins via its extracellular domain and generic role for the syndecan family of proteoglycans as signaling “hubs” at ECM adhesion sites [14]. Considering the nature of SDC1, we wonder what role this proteoglycan on the regulation of SASP and inflammaging mediated by miR-21.

## Material and methods

### Tissue collection

Human umbilical cord stromas were obtained from cesarean surgeries performed on healthy women; these tissues were obtained with fully informed consent and ethical approval by the supervision from the Clinical Experimentation Ethics Committee (CEEC # 2021/010). All surgeries were performed in the Maternity Facility at Complejo Hospitalario Universitario A Coruña.

### Isolation and characterization of MSC

MSCs were isolated from human umbilical stromal tissue using a protocol developed by our group [3]. Briefly, the isolation was performed through explants after three short incubations with a cocktail containing 1.2 U/mL dispase and 112 U/mL type I collagenase (all from Sigma-Aldrich). After 3 days, the explants were removed from the plate, leaving attached the MSC, which were cultured in monolayer in Dulbecco's modified Eagle's medium (DMEM) with 10% fetal bovine serum, 1% penicillin, and 1% streptomycin. The cells were used in the experiments when they reached 90% confluence. SSTN was dissolved in 1 mL of DMSO obtaining a stock solution of 305 µM. This stock mixed with DMEM 10% FBS to obtain the work concentrations 10 µM, 1 µM, 0.5 µM and 0.05 µM used in this work.

### Biological differentiation

Chondrogenic differentiation was performed using MSC at P1 which were seeded into 6-well plates (Sarstedt) at 2 × 10^5^ cells per well in DMEM with 15% knockout serum (Gibco, Invitrogen), 5 mg/mL ascorbic acid, 6 μg/mL transferrin, 10 μM dexamethasone, 1 × 10^−7^ M retinoic acid (all form Sigma-Aldrich), and 1 ng/mL recombinant human transforming growth factor-β3 (TGF-β3) (ProSpec-Tany TechnoGene; Deltaclon). Adipogenic and osteogenic differentiation were performed with MSCs at P1 seeded in 6-well plates (Sarstedt) at 2 × 10^5^ cells per well in adipogenic or osteogenic commercial medium (Cambrex, Lonza), following the manufacturer's instructions, to assess the mesodermal differentiation potential. Control MSC were grown under DMEM with 10% knockout serum (Gibco, Invitrogen), 1% penicillin, and 1% streptomycin (Sigma-Aldrich). All the mediums were changed every 3 days.

### Immunohistochemistry analysis

MSC following the different differentiations were stained with Hematoxylin-Eosin, Red Alizarin and Oil Red O after 21 days in culture (all from Sigma-Aldrich). All cultures were fixed in 10 mM sodium periodate, 2% paraformaldehyde, 75 mM l-lysine dihydrochloride, and 37.5 mM dibasic sodium phosphate (all from Sigma-Aldrich) at pH 7.4 for 15 min at room temperature and then air dried. The differentiated cells were stained with a filtered solution of 0.3% Oil Red O to reveal lipid droplets or with Alizarin Red S 2% aqueous solution at pH 4.2 (Sigma-Aldrich) for 3 min to assess calcium deposits.

### FACS analysis

The cells were washed twice with PBS and incubated for 1 h at RT with the following direct antibodies: phycoerythrin (PE) mouse anti-human CD34 (1:20; DakoCytomation); FITC mouse anti-human CD45 (1:20; BD Pharmingen); FITC mouse anti-human CD105 (1:100; Serotec); PE-Cy5.5-conjugated mouse anti-human CD90 (1:20; BD Pharmingen); and PE-conjugated anti-human CD73 (1:20; BD Pharmingen). The stained cells were then washed twice with PBS and 10 × 10^5^ cells were analyzed with a FACSAria flow cytometer (BD Bioscience). FACS data were generated by DIVA software (BD Bioscience). Negative control staining was performed using FITC-conjugated mouse IgG1k isotype, PE-conjugated mouse IgG1k isotype, and PE-Cy5.5-conjugated mouse IgG1k isotype (all from BD Pharmingen).

### EV measurement

EVs released from MSCs were isolated by ultracentrifugation processes [17]. Size distribution and concentration measurement were conducted on a second-generation nanoparticle tracking analysis (NTA) instrument, NanoSight LM12 using Nanoparticle Tracking Analysis 2.3 software (NanoSight Ltd., Amesbury, UK) based on Brownian motion of the particles. The temperature was controlled at 24 °C. The data acquisition parameters were set as follows: All parameters were recommended by the manufacturer for EV analysis. After initial wash and calibration, samples were diluted to particles per milliliter in PBS.

### Transmission electron microscopy

Purified EVs were covered with Formvar-carbon-coated EM grids to promote the absorption of EVs onto membranes over 20 min in a dry environment at room temperature. The grids were then placed directly on a drop of 1% glutaraldehyde and incubated for 5 min to remove the negative background. The grids were washed seven times with distilled water for 2 min each and examined using a Transmission Electron Microscope JEOL JEM-1010 (Jeol Ltd., Tokyo, Japan) at an acceleration voltage of 100 kV using a Megaview II high-resolution cooled digital camera.

### Permanent transduction of MSCs with miR-21 inhibitor

The Lenti-X™ Lentiviral Expression System (Clontech Laboratories Inc.) was used following the manufacturer's protocol. One day before transfection 4 × 10^6^ 293 T producer cells were placed on 100 mm plates in penicillin/streptomycin-free DMEM supplemented with 5% FBS. The following day, three different Polyethyleneimine (PEI)-based transfections were performed using miRZip-21 AntimicroRNA in SI505A-1 construct and the vectors psPAX2 and pMD2.G (all from SBI, System Biosciences, CA, USA) using PEI. The cells were incubated overnight with the transfection mixture, then washed with PBS and incubated with 8 mL of fresh complete growth medium. Viral supernatants were collected at 48 h, 60 h and 72 h following transfection, centrifuged, filtered to remove cell debris and stored at 4 °C until transduction.

MSC were plated in 100 mm plates at 6 × 10^6^ cells per plate. After one day, the cells were 70% confluent. The cells were incubated sequentially with the 48 h, 60 h, and 72 h viral supernatants for 12 h. Following the last transduction, the cells were washed and incubated with fresh growth medium to allow puromycin-resistance expression. Two days later, puromycin selection was performed by incubating the cells in growth media supplemented with 1 μg/mL puromycin (Clontech Laboratories Inc.) for five days. After selection, transduced cells were washed and allowed to recover in complete media for two days.

### miRNA transitory transfections

MSCs were incubated with 40 nM hsa-miR-21-5p miRVana™ miRNA mimic or 40 nM control negative miRVana™ miRNA mimic using the expression system following the manufacturer’s instructions. Validation by RT-PCR was done using Taqman®MicroRNA Assays following commercial instructions (all from Ambion, Applied Biosystems, Madrid, SP).

### Quantitative *shotgun* proteomic analysis

Three samples from MSC-miR-21^−^-derived EV and four samples from MSC-derived EV mimic were used to perform the shotgun analysis. The pellet containing EV was resuspended at 100 µL of RIPA buffer containing protease inhibitors and incubated in an orbital shaker for 30 min at 4 °C. Samples were then centrifugated at 16.000 rpm for 20 min at 4° C. The soluble protein fraction was centrifuged for 4 h at − 20 ºC in six volumes of cold acetone.

After centrifugation at 14000xG for 15 min at 4 °C the supernatants were carefully removed by inversion of the tube and the pellets were air-dried for thirty seconds. 100 µL of 0.1 M triethylammonium bicarbonate (TEAB) were added to the pellets, and the protein was completely dissolved by several cycles of vortexing and sonication. The concentration of protein was determined by BCA assay, and 20 µg from each sample were digested with trypsin (ratio 1:40) (16 h at 37 ºC) and labeled using Tandem Mass Tag™ (10-plex) (ThermoFisher, San Jose, USA) following the procedure from Morente-López et al. [17] The MSC-miR-21^−^-derived EV samples were labeled with tags 129N, 129C and 130N, whereas the MSC-mimic-derived EV samples were labeled with tags 126, 127N, 127C and 128N. The labeled peptides were fractionated using a Basic Reversed Phase fractionation kit (Thermo Fisher, Scientific, San Jose, USA).

### LC–MS/MS analysis (Q-exactive HF)

The eight different peptide fractions were analyzed by nano-liquid chromatography (nano Easy-nLC 1000, Thermo Fisher Scientific) coupled to a Q-Exactive HF high-resolution mass spectrometer (Thermo Fisher Scientific, Bremen, Germany). The peptides (1 µg per injection) were concentrated ¨on-line¨ by reverse phase chromatography (RP) using an Acclaim PepMap 100 guard column (Thermo Scientific, 20 mm × 75 µm ID, C18 of 3 µm particle diameter and 100 Å pore size) and then separated on a C18 Picofrit reversed phase analytical column (Thermo Scientific Easy Spray Column, PepMap RSLC C18 500 mm × 75 µm ID, 2 µm particle diameter, 100 Å pore size) with integrated spray tip, thermostatted, operating at a flow rate of 250 nL / min. The peptides were eluted using a gradient from 2 to 35% buffer B in 150 min and up to 40% in 10 min (Additional file 1: Fig. S1). The composition of the phases was as follows: buffer A: 0.1% of formic acid (FA) in water; Buffer B: 0.1% of FA in ACN.

The nano-HPLC was coupled on-line to the nanoelectrospray source of the Q-Exactive HF mass spectrometer with which the peptides were analyzed. The entry of the peptides was carried out by ionization with electrospray using the tip integrated in the analytical column.

The data acquisition was carried out with a voltage of 1.8 kV for the electrospray and the ´ion transfer tube´ that guides the ions from the spray to the interior of the mass spectrometer had a capillary temperature of 270 ºC.

The peptides were detected with a resolution of 120,000 in Full scan MS mode in a mass range m/z of 340–1600 Da. MS/MS data were acquired in MS data-dependent acquisition (DDA) mode. Thus, in each microscan up to 15 precursors with a load from 2+ to 4+ were selected, depending on their intensity (threshold: 2 × 10^4^), with dynamic exclusion of 20 s, followed by their isolation with a window width of ± 2 units of m/z, in a maximum time of 200 ms for its fragmentation by HCD (Higher-Energy Collision Dissociation) with a normalized collision energy of 32%. MS/MS spectra were acquired in positive mode (Additional file 2: Fig. S2).

### Reverse transcription quantitative PCR analysis

Total RNA from culture cells was isolated with TRIzol® reagent (Thermo Fisher Scientific, Waltham, MA, USA). For miRNA detection, cDNA was generated from DNaseI-treated RNA, using a QuantiMir RT Kit (System Biosciences, Palo Alto, CA, USA), according to the manufacturer’s instructions. PCR products were amplified using specific primers for miRNAs, miR-21-5p (rno481342_mir) and U6 (Rn01526055_g1) small nuclear RNA (Thermo Fisher Scientific, Waltham, MA, USA). The amplification program consisted of an initial denaturation at 50 °C for 2 min, followed by 95 °C for 10 min, and 50 cycles of annealing at 95 °C for 15 s and extension at 60 °C for 1 min. Primers for the amplification of rat genes are described in Table 1. The amplification program consisted of an initial denaturation at 92 °C for 2 min, followed by 40 cycles of annealing at 95 °C for 15 s; annealing at 55–62 °C, depending on the gene, for 30 s; and extension at 72 °C for 15 s. PCRs were done in triplicate, with each set of assays repeated three times. To minimize the effects of unequal quantities of starting RNA and to eliminate potential sources of inconsistency, relative expression levels of each gene were normalized to ribosomal protein (HPRT) or U6 small nuclear RNA using the 2 − ΔΔCT method [13]. Control experiments utilized no reverse transcriptase. TC28a2 Human Chondrocyte Cell Line (Sigma-Aldrich, St. Louis, USA) were cultured in monolayer in Dulbecco's modified Eagle's medium (DMEM) with 15% fetal bovine serum, 1% penicillin, and 1% streptomycin. These cells were used as a control cell line when they reached 90% confluence.

**Table 1:** Specific primers for real-time reverse transcriptase polymerase chain reaction (RT-PCR) amplification, listed with their annealing temperature (AT)

Gene name	Fw primer	Rv primer	mRNA ID	AT (°C)
*IL-6*	atgcctcacacggagactgt	aagtgggttgtttgcctttg	NM_005103.4	61
*HMGB*	ctcctggagggccaggaatc	atatacacaggccgatgtgg	NM_00510	61
*S100A6*	aggagaccttgcgaagacagg	gcggttgccacttgtttag	NM_001107248	61
*IL-1B*	gagcaaagtgcgtgaggagt	tccccctccttcttggtatt	NM_001002016.2	61
*S100A4*	atcaagcaagcgacatctca	caggccttggttaccagaaa	NM_019906.1	61
*TLR4*	gacgtagccattgtgaaggag	ccatcattcttgaggaggaagt	NM_033230.2	61
*HPRT*	agccgaccggttctgtcat	agccgaccggttctgtca	NM_012583.2	61

*Fw* forward, *Rv* reverse

### Immunoblot analysis

Immunoblot analysis was performed with 40 μg total protein extracted from MSCs or 20 μg total protein extracted from MSC-derived EVs. Proteins were separated according to their molecular weight using sodium dodecyl sulfate–polyacrylamide gel electrophoresis (SDS-PAGE), with the percentage (w/v) bis-acrylamide (Sigma-Aldrich, St. Louis, USA) of the resolving gels being determined by the size of the proteins. Proteins were then transferred to nitrocellulose membranes using a semi-dry method, using buffer with 20% (v/v) methanol (Panreac, Barcelona, Spain) for small proteins (< 100 kDa) or 10% (v/v) methanol (Panreac, Barcelona, Spain) for large proteins (> 100 kDa). Nitrocellulose membranes were then incubated for 1 h with agitation at room temperature in blocking buffer, consisting of 5% (w/v) bovine serum albumin (BSA) for phospho-proteins and 5% (w/v) milk (Sigma-Aldrich, St. Louis, USA). The membranes were probed with antibodies diluted in blocking buffer at 4 °C overnight. The following day, the membranes were washed three times for 5 min with Tris-buffered saline with 0.1% (v/v) Tween® 20 (TBST). The membranes were then incubated for 1 h at room temperature in horseradish peroxidase (HRP)-conjugated secondary antibodies diluted in blocking buffer. After that, the membranes were washed three times in TBST buffer for 5 min with agitation and twice using Tris-buffered saline (TBS) for 5 min with agitation. An Amersham ECL Western Blotting Analysis System (GE Healthcare, Little Chalfont, UK) was used to visualize protein-binding antibodies. The blots were probed with antibodies directed against TLR4 (Immnunostep, Salamanca, SP); phosphor-ERK1/2; ERK1/2; phosphor-AKT; AKT; SDC1 (all from Cell Signaling Technology, Beverly, MA, USA); Calnexin and CD63 (Abcam, Cambridge, MA, USA); Syntenin-1 and GM130 (all from Santa Cruz Biotech,

Heidelberg, GER) and β-actin (Sigma-Aldrich, St. Louis, MO, USA) or GAPDH (Cell Signaling Technology, Beverly, MA, USA) was housekeeping protein used as loading controls. Adequate concentrations for each antibody were determined empirically. Blot images were digitized using a LAS 3000 image analyzer (GE Healthcare, Little Chalfont, UK). Full-length blots/gels are presented in Additional file 3: Fig. S3. Densitometry analysis of band intensities was performed using ImageQuant 5.2 software (GE Healthcare, Little Chalfont, UK).

### Cell counting analysis

Cell viability was analyzed in cytotoxicity assays by Cell Counting Kit-8 following the manufacturer instructions. Briefly, MSC were seed in a 96-well plate at a density of 10^4^ cells/well in 100 μL of culture medium with or without different amounts of SSTN (10 µM, 1 µM, 0.5 µM, 0.05 µM) and incubate the plate for a different lengths of time (1, 2, 5 and 6 h) in the incubator. 10 μL of CCK-8 solution to each well of the plate was added, using a repeating pipettor. Incubate the plate for 4 h in the incubator and after shaking the absorbance was measured at 450 nm using a microplate reader.

### Crystal violet analysis

MSC obtained from umbilical cordon were plated in a 96-well 15,000 cells per well. Once the development time of the experiment is over, the medium is removed from the cells, they are washed with distilled water twice and they are fixed in the wells with 4% (w/v) paraformaldehyde (Sigma-Aldrich, Spain). After 5 min in rotation at room temperature, the fixative is then removed, they are washed again with distilled water and crystal violet 0.5% (v/v) (Sigma-Aldrich, Spain) is added for at least 30 min in rotation at room temperature. After this time, this dye is removed and allowed to dry. When dry, the plate is scanned. Subsequently, the stained crystal violet will be dissolved with 30% (v/v) acetic acid (Merck, Germany) for 30 min in rotation at room temperature. Finally, the plate is read in the NanoQuant spectrophotometer (Tecan infinite M200, United States) of absorbance at a wavelength of 590 nm.

### Luciferase assay

The Luciferase Reporter Assay System (E1910; Promega, Madison, WI, USA) was used to detect luciferase viability. A 62-bp fragment from the 3′ untranslated region (3′-UTR) of SDC1 (position 1636–1782, NM_002997.5) containing the miRNA-21 binding sites were cloned into the Bam HI site of the pGL4.14 [luc2/Hygro] luciferase reporter vector (Promega). The corresponding mutant construct was created by mutating the seed region of the miR-21 binding site. The constructs were then verified by sequencing. Using lipofectamine 2000 (Invitrogen), cells were transfected with the reporter constructs containing either the targeting sequence from the SDC1 3´-UTR (named pGL4.14-SDC1) or its mutant (named pGL4.14-SDC1m). The pRL-TK vector (Promega) was co-transfected as internal control for normalization of the transfection efficiency. The luciferase activities were then determined using a Tecan Infinite 200 Pro M Plex Microplate Reader- AV (Tecan, Mánnedorf, Suíza).

### Statistical treatment of the data

Raw files from quantitative shotgun proteomic analysis were processed using Proteome Discoverer 2.4 software (ThermoFisher, Madrid, SP). Modulation of the quantified proteins and statistical analysis of the modulation were done using the “TMT quantitation” and “statistical analysis” modules integrated in the search engine Proteome Discoverer 2.4 software (Thermo Fisher Scientific) against the 2020_01 UniProtKB/Swiss-Prot release (of 26-Feb-20 of containing 561,911 sequence entries). Identified results were validated for < 1% of false discovery rate (FDR).

All the results are expressed as the mean ± standard deviation. The association between qualitative variables with the chi-square test will be studied. We will compare the means of the variables studied between the different treatments after checking normality with the Kolmogorov–Smirnov test, with the T test for paired samples, the Wilcoxon test or two-way ANOVA test to determine the difference between treatments. All experiments were independently repeated three times. *P* < 0.05 was considered statistically significant. Statistical analysis was performed using GraphPad Prism 7 (GraphPad Software, Inc, San Diego, CA, USA).

## Results

### Characterization of MSCs from umbilical cord stroma and their EV

**Fig. 1:**
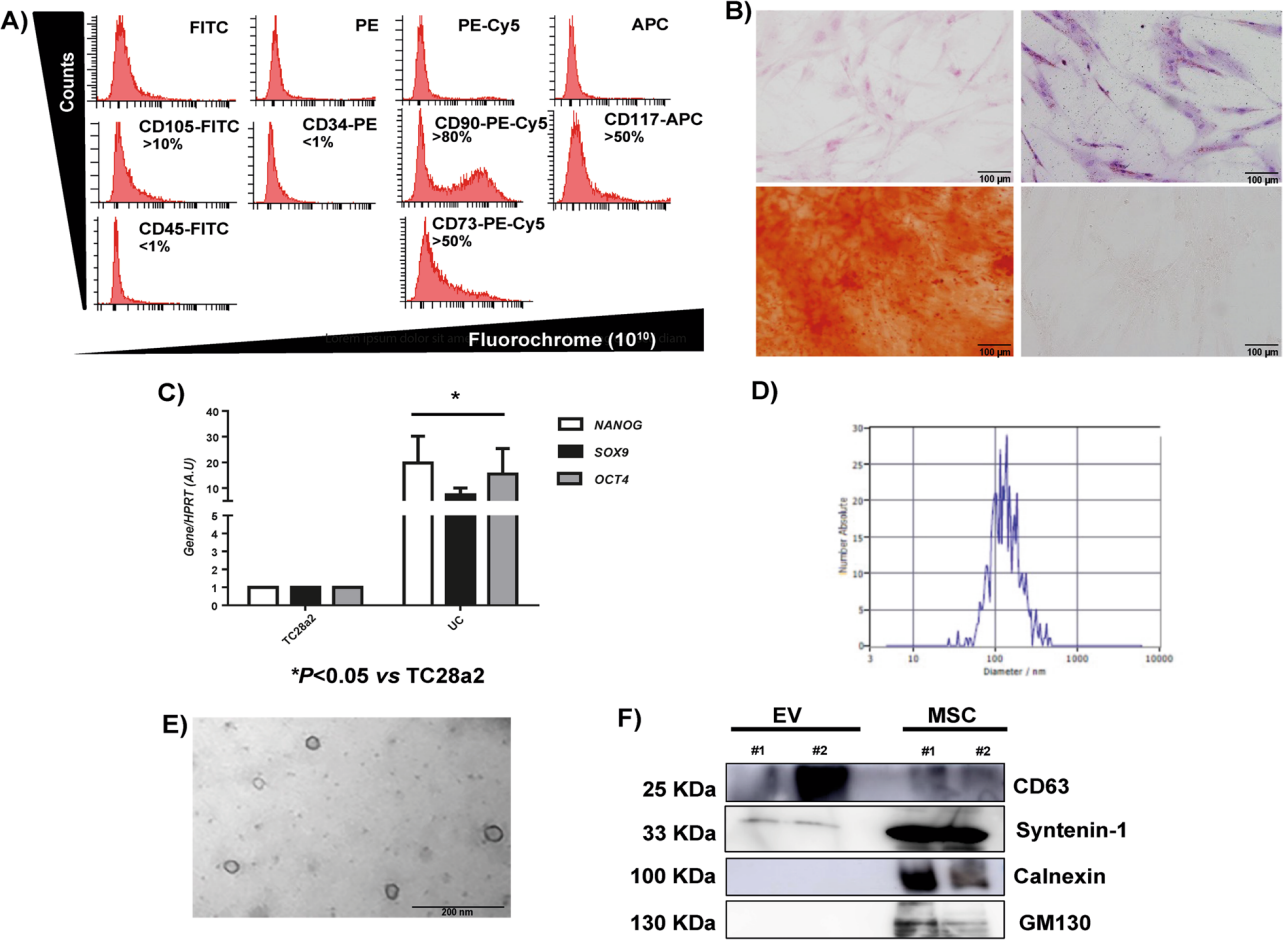
Characterization of MSCs from umbilical cord stroma and their EV. **A** One representative fluorescence-activated cell sorting (FACS) assay is shown. Positive MSC markers (CD105, CD90 and CD73) and negative hematopoietic markers (CD34 and CD45). **B** Representative pictures of immunohistochemical analysis (OR = Oil Red O, SafO = safranin O and AR = Alizarin Red from human umbilical cord stroma after 14 days with specific differentiation medium (DMEM, AD = Adipocyte medium, CH = Chondrocyte medium and OS = Osteocyte medium). **C** Histogram represents gene expression of pluripotency markers, Nanog, Sox9 and Oct4 in MSC in front of TC28a2 cell line. Real-time reverse transcriptase PCR (RT-qPCR) analysis normalized by expression of HPRT gene used as housekeeping. *P value less than 0.05 was considered statistically significant using two-way ANOVA test (n = 3). **D** Representative result from the NTA assay of MSC-derived EV. **E** Electron micrograph of MSC-derived EV (scale bar = 200 nm). **F** Immunoblot staining for exosome markers CD63 and Syntenin-1, Calnexin and GM130 as a negative control. Full-length blots/gels are presented in Additional file 3: Fig. S3

MSC isolated from human umbilical cord stroma were characterized by flow cytometry. In Fig. 1A is shown the levels of cells positive for CD45 and CD34, hematopoietic markers, less than 1%. These MSC were positives for more than 10% ± 5% for CD105, more than 80 ± 5% cells positive for CD90 and more than 50 ± 5% cells positive for CD73 and CD117 (Fig. 1A). Besides, biological characterization of MSC was performed, differentiating toward chondrocyte, adipocyte and osteocyte lines during 14 days in culture using specific media (Fig. 1B). Pluripotent MSC markers, *Nanog*, *Sox9* and *Oct4,* were measured by qRT-PCR, which were more significantly statistical expressed than in the TC28a2 line used as a control (Fig. 1C). The production of MSC-derived EV and their size was 160 ± 18 nm by NTA (Fig. 1D). Electron microscopy revealed MSC-derived EVs as small vesicles, typically 40–80 nm in diameter (Fig. 3E). Western analysis of EVs revealed the level of CD63 and Syntenin-1 (exosome membrane markers) and absence of Calnexin, (endoplasmic reticulum marker) and GM130 (Golgi apparatus marker) (Fig. 1F).

### Effect of miR-21 inhibition on SASP of MSC and characterization of MSC-miR-21^−^-derived EV

**Fig. 2:**
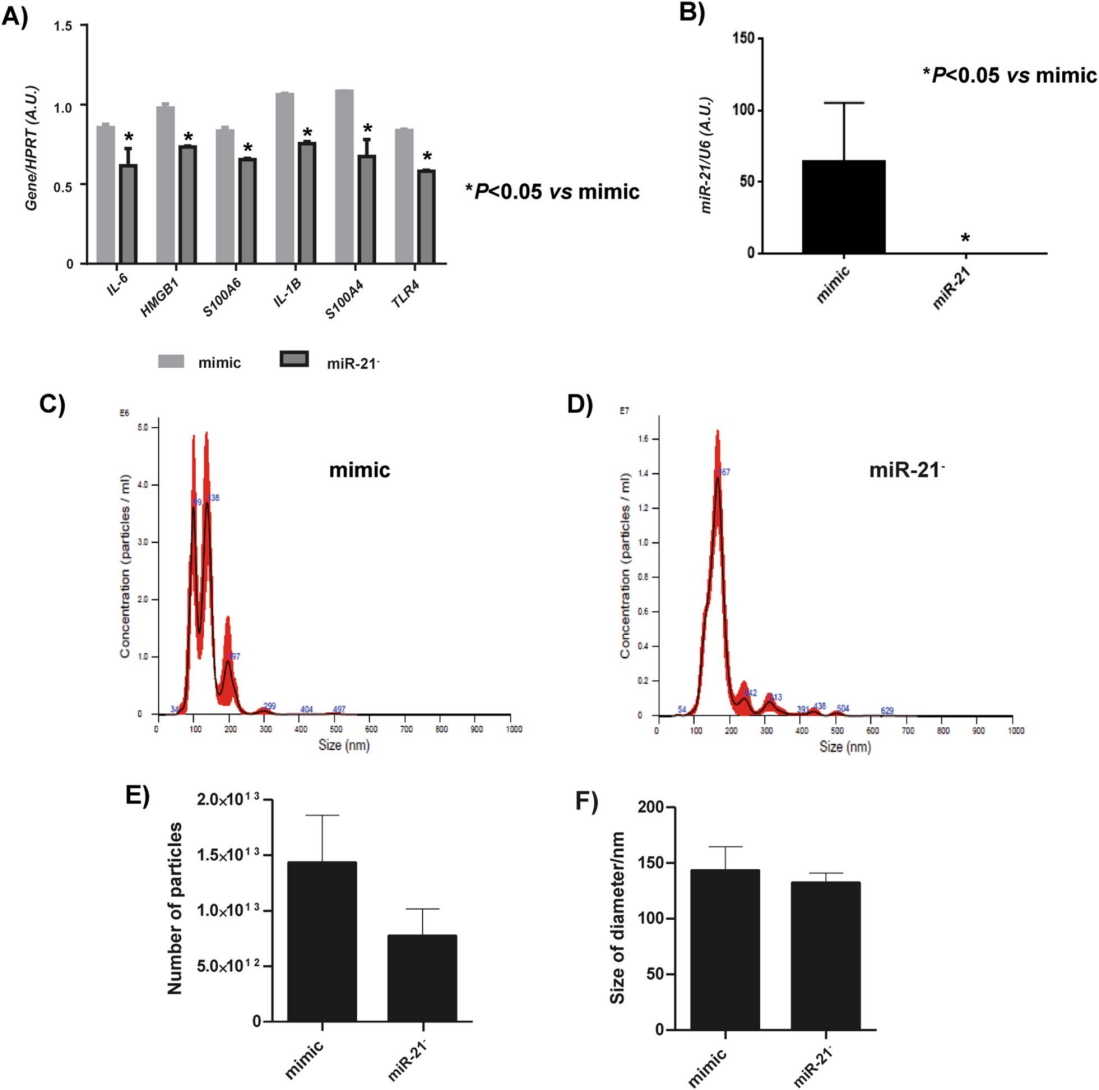
Effect of inhibition of miR21 and MSC-miR-21^−^-derived EV. **A** Histogram represents gene expression of SASP and inflammaging markers (S100A4, S100A6, HMGB1, IL-6, TLR4 and IL-1β) from MSC-mimic and MSC-miR-21^−^. Real-time reverse transcriptase PCR (RT-qPCR) analysis normalized by expression of HPRT gene used as housekeeping. **B** Representative result from the NTA assay of MSC-mimic-derived EV. **C** Representative result from the NTA assay of and MSC-miR-21^−^-derived EV. **D** Histogram represents gene expression of miR-21 in MSC-mimic and MSC-miR-21^−^. Real-time reverse transcriptase PCR (RT-qPCR) analysis normalized by expression of U6 gene used as housekeeping. **E** Histogram represents number of particles in MSC-mimic and MSC-miR21^−^ by NTA. **F** Histogram represents size of particles in MSC-mimic and MSC-miR21^−^ by NTA. Bars means ± SEM from three independent experiments.*P value less than 0.05 was considered statistically significant two-way ANOVA test (n = 3)

The analysis by RT-qPCR of SASP markers in MSC-miR-21^−^ indicated that all of them had decreased their expression statistically significative versus mimic (Fig. 2A). The expression of miR-21 in transfected MSC was checked by RT-qPCR through TaqMan probes, indicating that miR-21 was under-expressed in EV derived from MSC transfected with the miR-21 inhibitor compared to MSC-mimic (Fig. 2B). NTA revealed (Fig. 2C, D) that MSC-derived EV production was decreased (25%) in MSC with its miR-21 inhibited *versus* MSC-mimic-derived EV, this decrease was not statistically significant (Fig. 2E); besides the size of the extracellular vesicles was 140 ± 20 nm, which did not differ significantly between the transfected groups (Fig. 2F).

### Shotgun study of miR-21 effect on protein cargo of MSC-derived EV

Proteomic datasets are deposited and freely accessible at MassIVE repository (dataset MSV000086963 at www.massive.ucsd.edu).

**Fig. 3:**
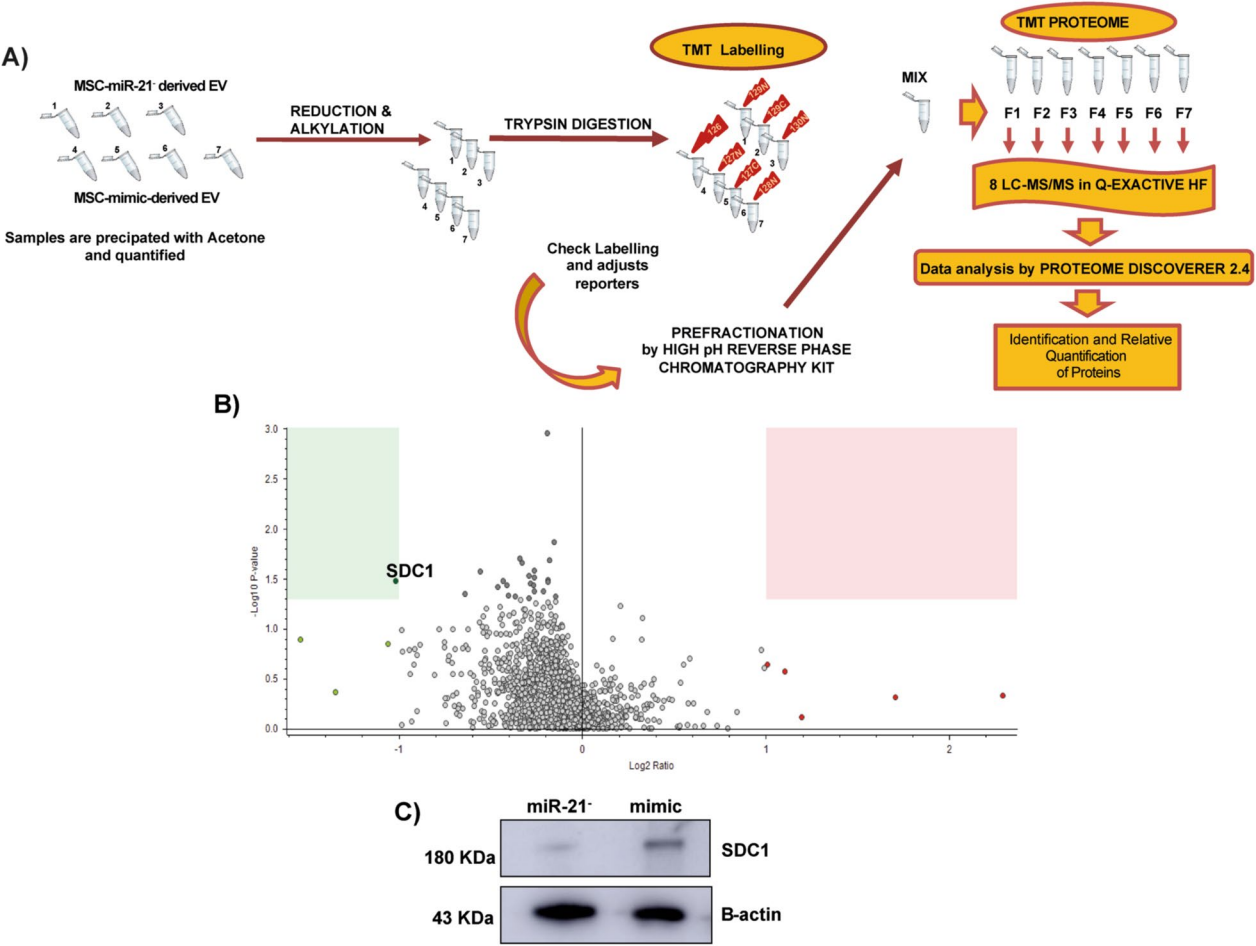
Shotgun study of miR21 in protein cargo of MSC-derived EV. **A** Workflow Peptides from MSC-mimic-derived EV and MSC-miR-21^−^-derived EV with 2 or 3 biological replicates were labeled with 7 TMT reporter from 10-plex reagents. These reagents are observed as a monoisotopic complex in the first round of MS analysis on a high-resolution mass spectrometer. During MS/MS and HCD-based fragmentation, the TMT labels peptides are fragmented to produce 8 reporter ions with distinguishable masses in the low m/z range, which allow relative protein quantitation based in their intensities ratios. **B** Volcano plot of all proteins identified in our shotgun study for the MSC-mimic and MSC-miR21^−^. SDC1 is the differentially expressed protein that was significantly downregulated by inhibition of miR-21 (fold change ≥ 2; *P* value ≤ 0.05). **C** Orthogonal validation of SDC1 in MSC-miR-21^−^ (miR-21.^−^), MSC-mimic (mimic) by western blot. B-actin was used as housekeeping. Full-length blots/gels are presented in Additional file 3: Fig. S3

A summary of the quantitative proteomics analysis is represented in Fig. 3B, which show the quantification results in this study represented in a volcano plot. A total of 1848 proteins were identified and quantified, 1141 of them with at least two unique peptides. Statistical analysis of the quantification results showed that SDC1 protein (Ensembl Gene ID: ENSG00000115884.10) level was significantly lower (fold change ≥ 2; *P* value ≤ 0.05) in the MSC-miR21^−^-derived EV than MSC-mimic-derived EV). The quality of SDC1 determination was demonstrated by total ion chromatogram (TIC) and extracted ion chromatogram (XIC) of the elution of the precursor (Additional file 1: Fig. S1), besides it was orthogonally validated by western blot (Fig. 3C).

### Paracrine effect of MSC- miR-21^−^-derived EV vs MSC-miR-21^+^-derived EV in vitro

**Fig. 4:**
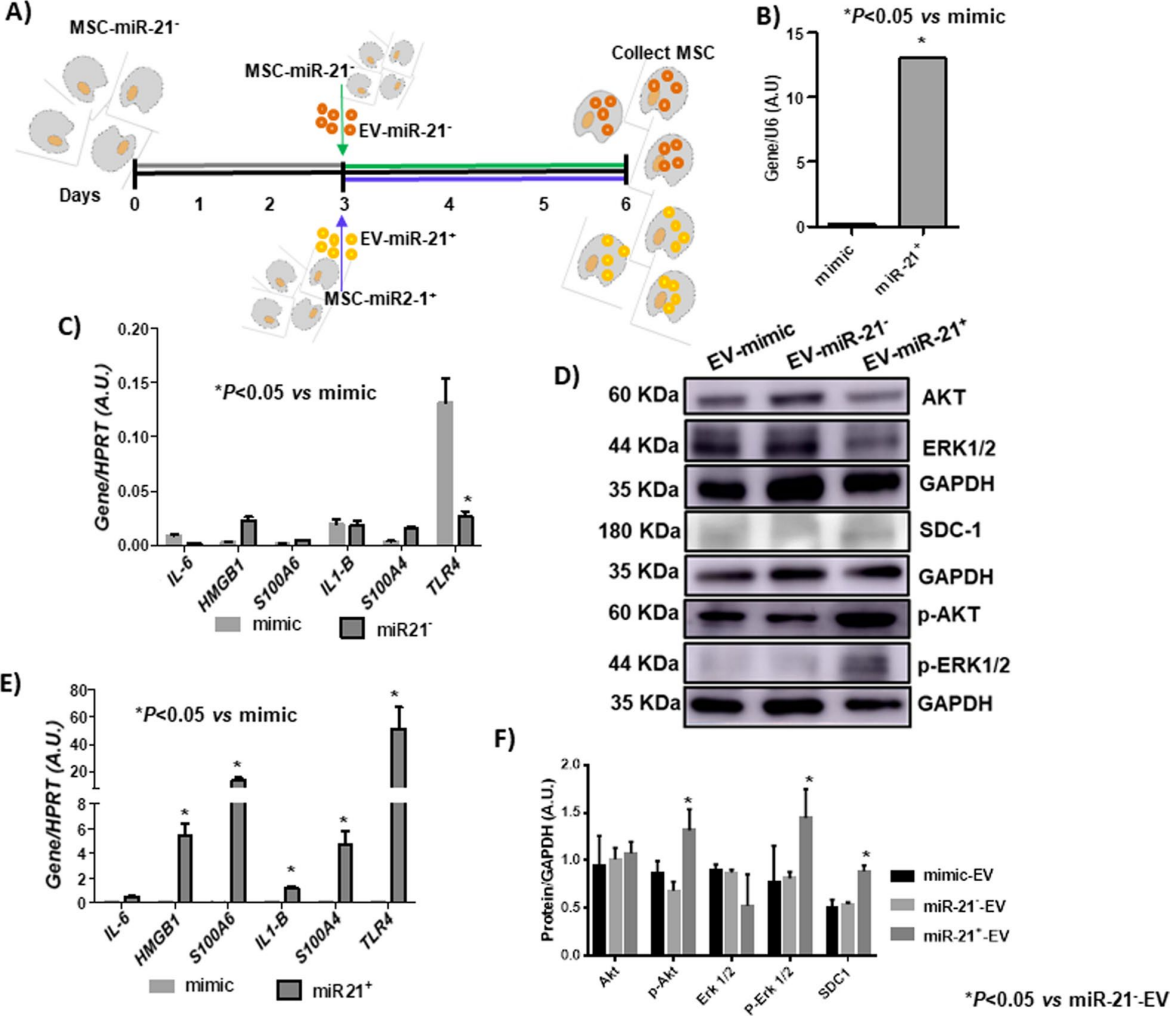
Paracrine effect of MSC-miR21^−^ -derived EV vs MSC-miR21^+^-derived EV in vitro. **A** Workflow of experiment using MSC-miR-21^−^-derived EV or MSC-miR-21^+^-derived EV to treat MSC-miR-21^−^ for 3 days. After that the cells were collected to performed the successive experiments. **B** Histogram represents gene expression of miR-21 in MSC-mimic and MSC-miR-21^+^ transitory transfected. Real-time reverse transcriptase PCR (RT-qPCR) analysis normalized by expression of *U6* gene used as housekeeping. *P value less than 0.05 was considered statistically significant. **C** Histogram represents gene expression of SASP and inflammaging markers (*S100A4, S100A6, HMGB1, IL-6, TLR4 and IL-1β)* from MSC-mimic and MSC-miR-21^−^ treated with MSC-miR-21^−^-derived EV. **D** Western blots analysis of MAPK family (ERK1/2, AKT, phosphor-ERK1/2, phosphor-AKT) and SDC1 from MSC-miR-21^−^ lysates described in the experiment above (**A**). Full-length blots/gels are presented in Additional file 3: Fig. S3. **E** Histogram represents gene expression of SASP and inflammaging markers (*S100A4, S100A6, HMGB1, IL-6, TLR4 and IL-1β*) from MSC-mimic and MSC-miR-21^−^ treated with MSC-miR-21^+^-derived EV. Real-time reverse transcriptase PCR (RT-qPCR) analysis normalized by expression of HPRT gene used as housekeeping. *P value less than 0.05 was considered statistically significant. **F** Densitometry results for normalized proteins relative to GAPDH. Bars are means ± SEM from three independent experiments. *P < 0.05 was considered statistically significant using two-way ANOVA test (n = 3)

We studied the paracrine effect of EV by culturing MSC-miR-21^−^ to which MSC-miR-21^−^-derived EV (2 × 10^7^ EV) or MSC-miR-21^+^-derived EV (2 × 10^7^ EV) were added (Fig. 4A). The increase of miR-21 in MSC-miR-21^+^-derived EV was verified by RT-PCR using TaqMan probes (Fig. 4B). The effect of these EV on SASP markers expression was studied by RT-PCR. MSC-miR21^−^-derived EV were found to produce a statistically significant inhibitory effect on SASP expression (Fig. 4C) and in the opposite way, these cells increased their SASP expression statistically significant when were treated with MSC-miR-21^+^-derived EV (Fig. 4E). Western blot was performed and it was observed that phosphorylated protein levels, Serine/threonine kinases (phosphor-ERK1/2 and phosphor-AKT), which acts as essential components of the MAP kinase signal transduction pathway, were statistically significant increased when MSC-miR-21^−^ were treated with MSC-miR-21^+^-derived EV (Fig. 4D–F).

### Role of SDC1 in EV behavior

**Fig. 5:**
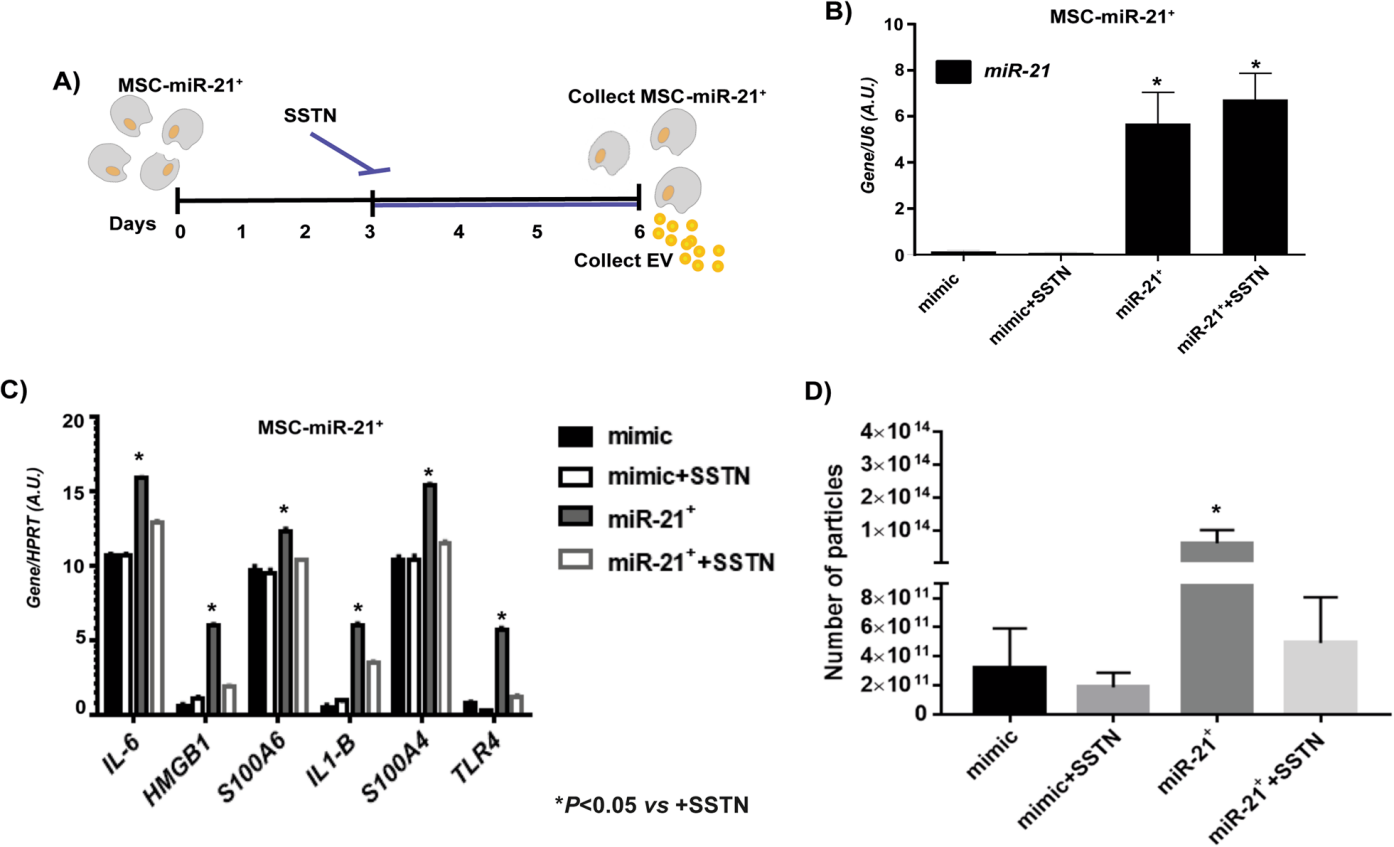
Validation of SDC1 in vitro. **A** Workflow of experiment using MSC-miR-21^+^ treated with SSTN during 3 days. After that the cells and their derived-EV were collected to performed the successive experiments. **B** Histogram represents gene expression of miR-21 in cells collected from workflow experiment described in (**A**). Real-time reverse transcriptase PCR (RT-qPCR) analysis normalized by expression of *U6* gene used as housekeeping. **C** Histogram represents gene expression of SASP and inflammaging markers (*S100A4, S100A6, HMGB1, IL-6, TLR4, IL-1β*) from cells collected from workflow experiment described in (**A**). Real-time reverse transcriptase PCR (RT-qPCR) analysis normalized by expression of HPRT gene used as housekeeping. **D** Histogram represents number of particles from cells collected from workflow experiment described in (**A**). Bars are means ± SEM from three independent experiments. *P value less than 0.05 was considered statistically significant using two-way ANOVA test (n = 3)

**Fig. 6:**
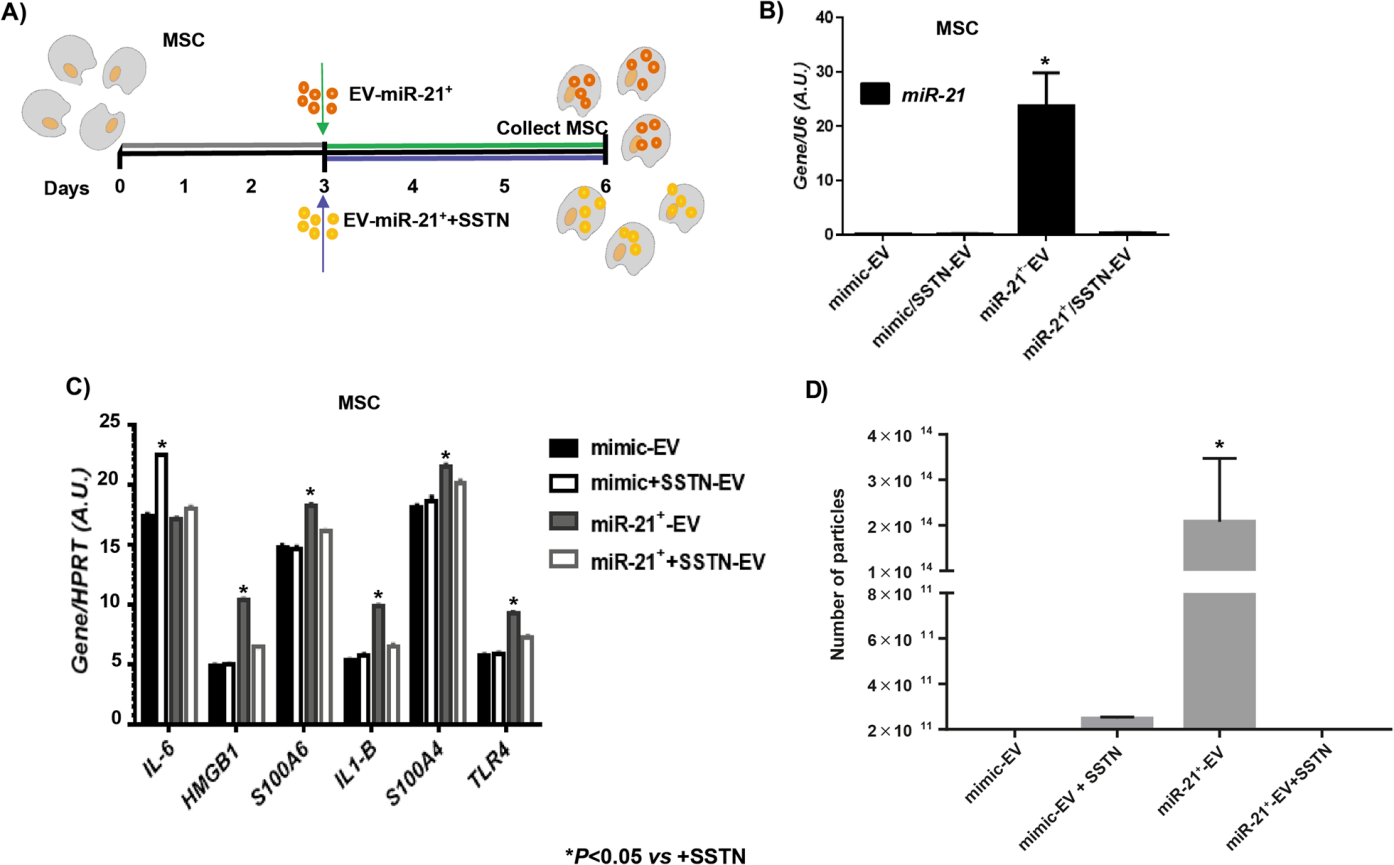
Paracrine validation of SDC1 in vitro. **A** Workflow of experiment using MSC treated with MSC-miR-21^+^-derived EV or MSC-miR-21^+^-derived EV previously treated with SSTN, for 3 days. After that the cells were collected to perform the successive experiments. **B** Histogram represents gene expression of miR-21 in cells collected from workflow experiment described in (**A**). Real-time reverse transcriptase PCR (RT-qPCR) analysis normalized by expression of U6 gene used as housekeeping. **C** Histogram represents gene expression of SASP and inflammaging markers (*S100A4, S100A6, HMGB1, IL-6, TLR4 and IL-1β*) from cells collected from workflow experiment described in (**A**). **D** Histogram represents number of particles from cells collected from workflow experiment described in (**A**). Bars are means ± SEM from three independent experiments. *P value less than 0.05 was considered statistically significant using two-way ANOVA test (n = 3)

The role of SDC1 in SASP and EV production was determined using its inhibitor, Synstatin (SSTN) (Fig. 5A). Firstable the adequate concentration of SSTN on MSC was checked and neither concentration of SSTN nor time incubation affected the viability of MSC through counting cell and crystal violet assays and *SDC1* was verified as the target gene for miR-21. Online target prediction was used to determine whether miR-21 can directly regulate SDC1, and the results were confirmed by luciferase reporter assay. The results identified a specific binding region between the *SDC1* gene and the miR-21 sequence, indicating that *SDC1* was the target gene of miR-21 (Additional file 2: Fig. S2). To prove that miR-21 affects the target site, WT and MUT sequences lacking the miR-21 combination sites in the SDC1 3′-UTR area were inserted using a reporter plasmid. The miR-21 mimic, *SDC1-WT* or *SDC1-MUT* recombined plasmids were transfected into the MSCs, and the results revealed that incubation with the CMM-miR-21^+^-derived EV had no significant effects on luciferase activity in the *SDC1-MUT* transfected MSC, whereas the luciferase activity was significantly increased following transfection with *SDC1-WT* in MSC incubated with CMM-miR-21 + -derived EV (*P* < 0.05). These results indicate that miR-21 may directly target SDC1. MSC that transiently over-expressed miR-21 was cultured for 3 days and 0.5 µM of SSTN was added to the medium to find out if the inhibition of SDC1 produced any effect on SASP through miR-21. The transient over-expression of miR-21 was validated by RT-qPCR and was statistically significant increased in the MSC-derived EV produced regardless of the SSTN in the medium (Fig. 5B). The expression of SASP markers was increased in the MSC-miR-21^+^ in a statistically significant way when were treated with MSC-miR-21^+^-derived EV but these increases were not produced with MSC-miR-21^+^ -derived EV plus SSTN treatment (Fig. 5C). The production of EV was observed to be statistically significant increased in the transduced over-expressing miR-21 MSC; however, this production was significantly reduced when SSTN was incorporated into the medium (Fig. 5D). The role of SSTN in paracrine effect of EV is graphical resume in Fig. 6A, when MSCs were treated with MSC-miR-21^+^-derived EV plus SSTN, the increase of miR-21 did not occur as did EVs produced by MSCs over-expressing miR-21 (MSC-miR-21^+^-derived EV) without SSTN (Fig. 6B). It was observed how SASP markers increased their expression in a statistically significant way in the MSCs treated with MSC-miR-21^+^-derived EV but these increases were not produced in the MSC treated with MSC-miR-21^+^ -derived EV plus SSTN (Fig. 6C). The production of EV was observed to be statistically significant increased in the MSCs transfected with the over-expressing miR-21-EV; however, this production was significantly reduced when SSTN was incorporated into the medium (Fig. 6D).

## Discussion

MSC from human umbilical cord stroma expressed the characteristic markers of stem cells recognized by the ISSCR [11, 23, 29] in addition to being susceptible to be differentiated into three different mesodermal lines when they are differentially cultured in the laboratory, and these cells showed a high expression of pluripotency genes. Afterward, their EVs showed expression of EV markers and an adequate size according to the latest International Society for Extracellular Vesicles (ISEV) specifications [27, 31] (Fig. 1). Several authors already indicated the role of miR-21 as an inflammation marker in age-associated diseases [20, 21] which was characterized by a prominent activation of the innate immune response. Besides elevated circulatory miR-21 concentrations have a positive correlation with aging [2]. Our group have reported that miR-21 was abundant in extracellular vesicles from aging-MSC and after conducting functional experiments inhibiting its expression, it was demonstrated the link between miR-21 expression and damage-associated molecular patterns and SASP by aging [9]. Our current results indicated that inhibition of miR-21 induced statistically significant decrease in the expression levels of SASP and inflammaging markers. After checking that miR-21 was effective inhibited in MSC-miR-21^−^ by RT-qPCR, it was checked its effect on number and size of derived-EV. The inhibition of miR-21 produced a decrease of number of derived-EV although it did not affect their size. Being further evidence on how the inhibition of miR-21 produces a decrease in SASP and EV production by MSC (Fig. 2).

A shotgun proteomic study was performed to decipher which proteins were affected in EV derived from MSC-miR-21^−^ (Fig. 3). Surprisingly, although they were detected 1848 proteins only SDC1 was statistically significant, this may be due to the fact that the MSCs from the human umbilical cord stroma have a lot of variability because of different ages from the donors [4]. Syndecans are integral membrane proteoglycans, they are characterized by the similarity of transmembrane and cytoplasmic domains of the central proteins and they may regulate cell behavior by participating in growth factor binding and matrix recognition [10]. SDC1 level is associated with salivary gland function and inflammation in patients with Sjögren's syndrome and it is increased in saliva [12]. Exploring the downstream target genes of miR-21-5p in TargetScan (http://www.targetscan.org/vert_72/), there is a putative common outcome was predicted with SDC1 (Additional file 2: Fig. S2).

Il-6 and IL-1β are recognized markers of SASP and cell inflammaging [15]. Both are canonically upregulated in cells entering senescence, increase with age and play a causal role in inflammaging and age-related diseases [30]. In senescence cells, HMGB1 leaves the nucleus to the cytoplasm and when secreted stimulates NF-κB activity through Toll-like receptor signaling. Its relocalization and secretion control the SASP of the cells. HBGB1 is an important paracrine contributor both in vitro and in vivo [1]. The success of the transitory transfection of miR-21 agonist on MSC was checked by RT-qPCR. Paracrine experiments were performed with their MSC-miR-21 + -derived EV to discern the influence of miR-21 on SASP. Our results indicated that SASP markers increased their expression statistically significantly when MSC-miR-21^−^ cells were treated with MSC-miR-21^+^-derived EV, and the opposite happened when the MSC-miR-21^−^ cells were treated with MSC-miR-21^−^-derived EV (Fig. 4, Additional file 3: Fig. S3). All our results are consistent with those published by Yang et al. on miR-21 prevents excessive inflammation after myocardial infarction [32] and participates in balancing the inflammatory response in infected decidua [6]. It was observed that overexpression of miR-21 activated of Toll-Like receptor 4 (TLR4) together with expression changes in the SASP [9]. SASP has a physiological role inside but acquire additional functions when exposed to the extracellular environment, and they are secreted or displayed by living cells undergoing stress. Arufe et al. published that MSC-miR-21^−^-derived EV as well as MSCs-miR-21^−^ improved the systemic inflammation through inactivation of ERK1/2 pathway in OA in vivo model [18]. ERK1/2 is an important messenger for extracellular and intracellular signals, playing a key role in numerous processes, including differentiation, proliferation and cellular senescence [26, 33]. Our results confirmed that over-expression of miR-21 was affecting the ERK1/2-AKT pathway increasing their phosphorylation in MSC in vitro, which is in line with those published by Shi et al. whose results indicated that miR-21 increased the phospho-Akt (p-Akt) level promoting proliferation in c-kit^+^ cardiac stem cells [24].

To assess the relationship between miR-21 and SDC1, functional studies were performed using the SDC1 inhibitor, SSTN [25]. SSTN is a selective inhibitor of the SDC1 and its sequence is unique to SDC1 and is not found in other syndecans or in any other known protein [22]. SSTN did not influence miR-21 expression levels of MSC-miR-21^+^-derived EV. However, SSTN prevented that MSC- miR-21^+^-derived EV from increasing miR-21 expression in recipient MSC cells. So the over-expression of all these markers is reduced in the MSC-miR-21^+^ treated with SSTN. Expression levels of SASP and inflammaging markers also decrease when MSCs are treated with MSC-miR-21^+^-derived EV previously treated with SSTN. In addition, SDC1 is influencing the effect of miR-21 on the expression of SASP and inflammaging markers, because of when SSTN is in the medium, it is blocking SDC1 (Fig. 5). Chen et al. reported a similar mechanism of SDC1 regulation through miR-665 in a vascular smooth muscle cell senescence [7].

Finally, we verified that blockade of SDC1 by SSTN reduces the EV-producing effect of miR-21 overexpression (Fig. 6). McNeill et al. published that over-expression of miR-21 induced the production of EV [16], as confirmed in our work, stating for the first time that this action is mediated by SDC1 since it does not occur when the SDC is blocked by SSTN.

## Conclusions

This work demonstrates the importance of miR-21 on the density of EV production and its role in inflammaging through SDC1. Besides, miR-21 exerted its inflammatory action on the ERK1/2-dependent MAPK pathway. All these results would indicate that SDC1 could be a new target in anti-inflammatory therapy.

### Supplementary Information

**Additional file 1: Fig. S1.** Quality check Q-Exactive HF from shotgun proteomic analysis. **A** Overlay of the Total Ion Chromatograms (TICs) of the eight different reversed phase fractions showing the elution profile along the 3 h of gradient. No saturation of the signal is detected and a progressive shift from more hydrophilic to more hydrophobic peptides can be observed in the fractions indicating a proper reversed phase fractionation. b Total Ion Chromatogram of the Fraction 2 showing the retention time (red bar) in which the SDC1 peptide was isolated and extracted Ion Chromatogram (XIC) of the elution of the precursor (top). Detailed view of both TIC and XIC (bottom). **C** Fragmentation spectrum of the SDC1 precursor. **D** Normalized abundances (blue: grouped; gray: individual) of the SDC1 precursor in mimic and miR21^−^ samples after processing with Proteome Discoverer 2.4 software.

**Additional file 2: Fig. S2.** SSTN Effectiveness. **A** Binding sites between SCD1 and miR-21 carried out using the http://www.targetscan.org/. **B** Relative luciferase activity was increased following treatment with a combination of mimic-EV or miR-21^+^-EV on MSC transfected with *SDC1-WT* or *SDC1-MUT* suggesting that miR-21 regulates SDC1. *P < 0.05 versus mimic treatment. The data are presented as the means ± EEM, analyzed by independent sample t test. The experiment was independently repeated 3 times. **C** SDC1 sequence by UniProt ID. In red the identified by our shotgun proteomic study and in underlined red SSTN sequence. **D** Representative plate of MSC stained crystal violet. **E** Crystal violet analysis of MSC treated with different amounts of SSTN (10 µM, 1 µM, 0.5 µM, 0.05 µM) to different times (1, 2, 5 and 6 h). **F** Cytotoxicity assays of MSC treated with different amounts of SSTN (10 µM, 1 µM, 0.5 µM, 0.05 µM) to different times (1, 2, 5 and 6 h).

**Additional file 3: Fig. S3.** Full-length blots/gels presented in the manuscript.

## Data Availability

The datasets presented in this study can be found in online repositories. The names of the repository/repositories and accession number(s) can be found at: http://massive.ucsd.edu/ProteoSAFe/status.jsp?task=7de2f5d81265401cbb8796f290d6baa6.

## Abbreviations

CCK-8: Cell counting kit 8
DMEM: Dulbecco's modified Eagle's medium
EV: Extracellular vesicles
MSC: Mesenchymal stem cells
LC-MS/MS: Liquid Chromatography-Mass/Mass spectrometry
OA: Osteoarthritis
SASP: Senescence-Associated Secretory Phenotype
SDC1: Syndecan 1
SSTN: Synstatin
TMT: Tandem Mass Tag

## References

[1] Acosta JC, Banito A, Wuestefeld T, Georgilis A, Janich P, Morton JP, Athineos D, Kang TW, Lasitschka F, Andrulis M, Pascual G, Morris KJ, Khan S, Jin H, Dharmalingam G, Snijders AP, Carroll T, Capper D, Pritchard C, Inman GJ, Longerich T, Sansom OJ, Benitah SA, Zender L, Gil J (2013). A complex secretory program orchestrated by the inflammasome controls paracrine senescence. Nat Cell Biol.

[2] Ali Sheikh MS (2022). The mir-21 inhibition enhanced HUVEC cellular viability during hypoxia-reoxygenation injury by regulating PDCD4. Mediators Inflamm.

[3] Arufe MC, De la Fuente A, Mateos J, Fernandez P, Rendal E, Diaz S, Fuentes I, De Toro FJ, Blanco FJ (2010). Secretome analysis of mesenchymal stem cells from human umbilical cord strome during the chondrogenesis. Osteoarthr Cartil.

[4] Babenko VA, Silachev DN, Danilina TI, Goryunov KV, Pevzner IB, Zorova LD, Popkov VA, Chernikov VP, Plotnikov EY, Sukhikh GT, Zorov DB (2021). Age-related changes in bone-marrow mesenchymal stem cells. Cells.

[5] Basisty N, Kale A, Jeon OH, Kuehnemann C, Payne T, Rao C, Holtz A, Shah S, Sharma V, Ferrucci L, Campisi J, Schilling B (2020). A proteomic atlas of senescence-associated secretomes for aging biomarker development. PLoS Biol.

[6] Castro-Leyva V, Arenas-Huertero F, Espejel-Núñez A, Giono Cerezo S, Flores-Pliego A, Espino S, Sosa Y, Reyes-Muñoz E, Vadillo-Ortega F, Borboa-Olivares H, Camacho-Arroyo I, Estrada-Gutierrez G (2022). miR-21 differentially regulates IL-1β and IL-10 expression in human decidual cells infected with streptococcus B. Reprod Biol.

[7] Chen T, Liang Q, Xu J, Zhang Y, Mo L, Zhang L (2021). MiR-665 regulates vascular smooth muscle cell senescence by interacting with LncRNA GAS5/SDC1. Front Cell Dev Biol.

[8] Dal Collo G, Adamo A, Gatti A, Tamellini E, Bazzoni R, Takam Kamga P, Tecchio C, Quaglia FM, Krampera M (2020). Functional dosing of mesenchymal stromal cell-derived extracellular vesicles for the prevention of acute graft-versus-host-disease. Stem Cells.

[9] Fafián-Labora J, Lesende-Rodriguez I, Fernández-Pernas P, Sangiao-Alvarellos S, Monserrat L, Arntz OJ, Loo FJ, Mateos J, Arufe MC (2017). Effect of age on pro-inflammatory miRNAs contained in mesenchymal stem cell-derived extracellular vesicles. Sci Rep.

[10] Hilgers K, Ibrahim SA, Kiesel L, Greve B, Espinoza-Sánchez NA, Götte M (2022). Differential impact of membrane-bound and soluble forms of the prognostic marker syndecan-1 on the invasiveness, migration, apoptosis, and proliferation of cervical cancer cells. Front Oncol.

[11] Hyun I, Lindvall O, Ahrlund-Richter L, Cattaneo E, Cavazzana-Calvo M, Cossu G, De Luca M, Fox IJ, Gerstle C, Goldstein RA, Hermerén G, High KA, Kim HO, Lee HP, Levy-Lahad E, Li L, Lo B, Marshak DR, McNab A, Munsie M, Nakauchi H, Rao M, Rooke HM, Valles CS, Srivastava A, Sugarman J, Taylor PL, Veiga A, Wong AL, Zoloth L, Daley GQ (2008). New ISSCR guidelines underscore major principles for responsible translational stem cell research. Cell Stem Cell.

[12] Lee NY, Kim NR, Kang JW, Kim G, Han MS, Jang JA, Ahn D, Jeong JH, Han MH, Nam EJ (2022). Increased salivary syndecan-1 level is associated with salivary gland function and inflammation in patients with Sjögren's syndrome. Scand J Rheumatol.

[13] Livak KJ, Schmittgen TD (2001). Analysis of relative gene expression data using real-time quantitative PCR and the 2(-Delta Delta C(T)) Method. Methods.

[14] Manon-Jensen T, Itoh Y, Couchman JR (2010). Proteoglycans in health and disease: the multiple roles of syndecan shedding. FEBS J.

[15] Matacchione G, Gurău F, Silvestrini A, Tiboni M, Mancini L, Valli D, Rippo MR, Recchioni R, Marcheselli F, Carnevali O, Procopio AD, Casettari L, Olivieri F (2021). Anti-SASP and anti-inflammatory activity of resveratrol, curcumin and β-caryophyllene association on human endothelial and monocytic cells. Biogerontology.

[16] McNeill B, Ostojic A, Rayner KJ, Ruel M, Suuronen EJ (2019). Collagen biomaterial stimulates the production of extracellular vesicles containing microRNA-21 and enhances the proangiogenic function of CD34. FASEB J.

[17] Morente-López M, Fafián-Labora JA, Carrera M, de Toro FJ, Gil C, Mateos J, Arufe MC (2021). Mesenchymal stem cell-derived extracellular vesicle isolation and their protein cargo characterization. Methods Mol Biol.

[18] Morente-López M, Mato-Basalo R, Lucio-Gallego S, Silva-Fernández L, González-Rodríguez A, De Toro FJ, Fafián-Labora JA, Arufe MC (2022). Therapy free of cells vs human mesenchymal stem cells from umbilical cord stroma to treat the inflammation in OA. Cell Mol Life Sci.

[19] Munk R, Panda AC, Grammatikakis I, Gorospe M, Abdelmohsen K (2017). Senescence-associated microRNAs. Int Rev Cell Mol Biol.

[20] Olivieri F, Spazzafumo L, Santini G, Lazzarini R, Albertini MC, Rippo MR, Galeazzi R, Abbatecola AM, Marcheselli F, Monti D, Ostan R, Cevenini E, Antonicelli R, Franceschi C, Procopio AD (2012). Age-related differences in the expression of circulating microRNAs: miR-21 as a new circulating marker of inflammaging. Mech Ageing Dev.

[21] Prabowo AS, van Scheppingen J, Iyer AM, Anink JJ, Spliet WG, van Rijen PC, Schouten-van Meeteren AY, Aronica E (2015). Differential expression and clinical significance of three inflammation-related microRNAs in gangliogliomas. J Neuroinflamm.

[22] Rapraeger AC (2013). Synstatin: a selective inhibitor of the syndecan-1-coupled IGF1R-αvβ3 integrin complex in tumorigenesis and angiogenesis. FEBS J.

[23] Rooke H (2006). The International Society for Stem Cell Research (ISSCR): history and perspectives. Regen Med.

[24] Shi B, Deng W, Long X, Zhao R, Wang Y, Chen W, Xu G, Sheng J, Wang D, Cao S (2017). miR-21 increases c-kit. PeerJ.

[25] Stueven NA, Beauvais DM, Hu R, Kimple RJ, Rapraeger AC (2023). Inhibiting IGF1R-mediated survival signaling in head and neck cancer with the peptidomimetic SSTN. Cancer Res Commun.

[26] Sun Y, Liu WZ, Liu T, Feng X, Yang N, Zhou HF (2015). Signaling pathway of MAPK/ERK in cell proliferation, differentiation, migration, senescence and apoptosis. J Recept Signal Transduct Res.

[27] Théry C, Witwer KW, Aikawa E, Alcaraz MJ, Anderson JD, Andriantsitohaina R, Antoniou A, Arab T, Archer F, Atkin-Smith GK, Ayre DC, Bach JM, Bachurski D, Baharvand H, Balaj L, Baldacchino S, Bauer NN, Baxter AA, Bebawy M, Beckham C, Bedina-Zavec A, Benmoussa A (2018). Minimal information for studies of extracellular vesicles 2018 (MISEV2018): a position statement of the International Society for Extracellular Vesicles and update of the MISEV2014 guidelines. J Extracell Vesicles.

[28] Ti D, Hao H, Fu X, Han W (2016). Mesenchymal stem cells-derived exosomal microRNAs contribute to wound inflammation. Sci China Life Sci.

[29] Turner L (2021). ISSCR's guidelines for stem cell research and clinical translation: supporting development of safe and efficacious stem cell-based interventions. Stem Cell Rep.

[30] Wei J, Xu H, Davies JL, Hemmings GP (1992). Increase of plasma IL-6 concentration with age in healthy subjects. Life Sci.

[31] Witwer KW, Van Balkom BWM, Bruno S, Choo A, Dominici M, Gimona M, Hill AF, De Kleijn D, Koh M, Lai RC, Mitsialis SA, Ortiz LA, Rohde E, Asada T, Toh WS, Weiss DJ, Zheng L, Giebel B, Lim SK (2019). Defining mesenchymal stromal cell (MSC)-derived small extracellular vesicles for therapeutic applications. J Extracell Vesicles.

[32] Yang L, Wang B, Zhou Q, Wang Y, Liu X, Liu Z, Zhan Z (2018). MicroRNA-21 prevents excessive inflammation and cardiac dysfunction after myocardial infarction through targeting KBTBD7. Cell Death Dis.

[33] Zou J, Lei T, Guo P, Yu J, Xu Q, Luo Y, Ke R, Huang D (2019). Mechanisms shaping the role of ERK1/2 in cellular senescence (review). Mol Med Rep.

