# Supplementary figures and images for: Effect of miR-21 in mesenchymal stem cells-derived extracellular vesicles behavior
Source: Stem Cell Res Ther. 2023 Dec 21;14:383. doi: 10.1186/s13287-023-03613-z (PMC10740217; doi:10.1186/s13287-023-03613-z)

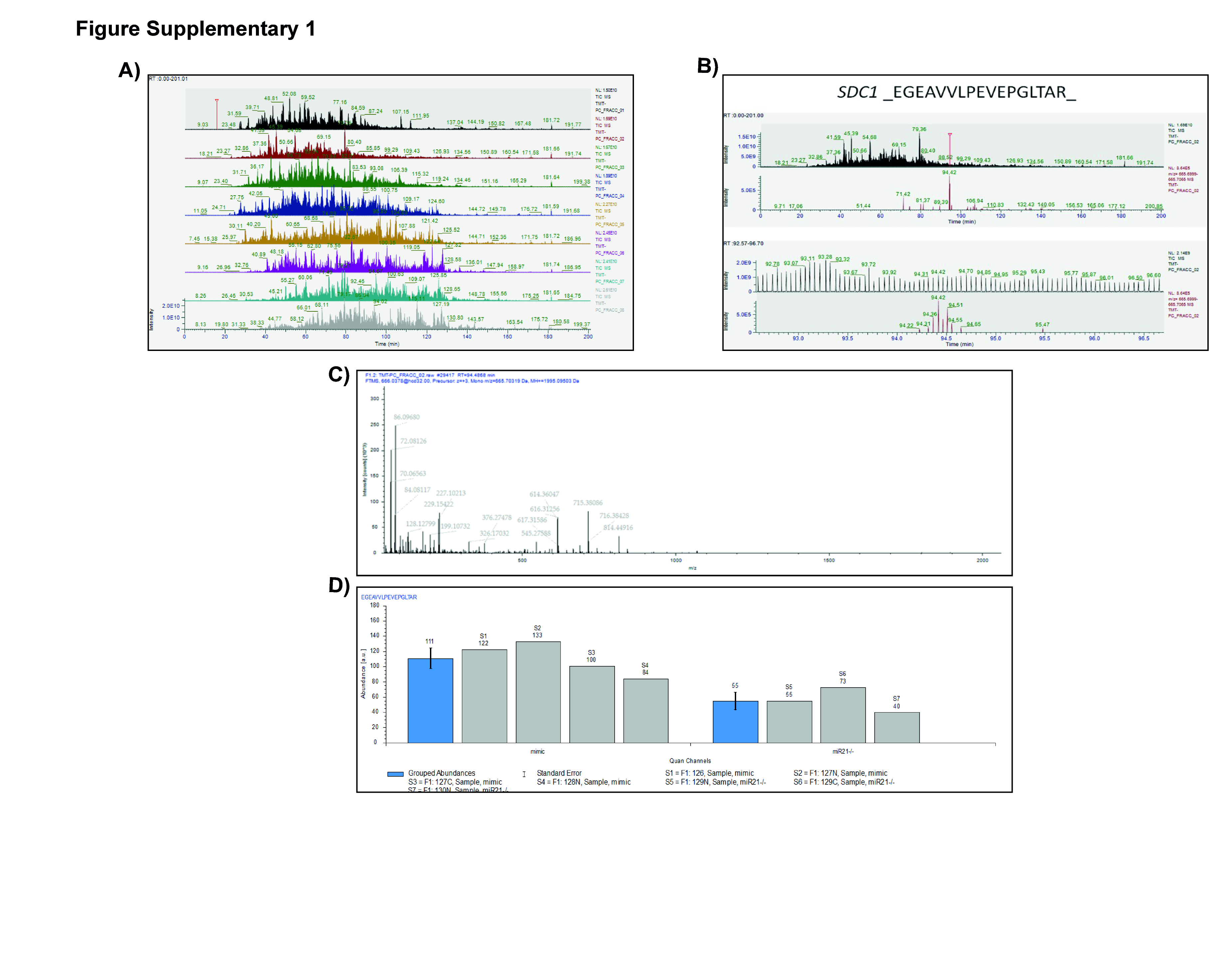

Supplement: Supplementary file 1 — Additional file 1: Fig. S1. Quality check Q-Exactive HF from shotgun proteomic analysis. A Overlay of the Total Ion Chromatograms (TICs) of the eight different reversed phase fractions showing the elution profile along the 3 h of gradient. No saturation of the signal is detected and a progressive shift from more hydrophilic to more hydrophobic peptides can be observed in the fractions indicating a proper reversed phase fractionation. b Total Ion Chromatogram of the Fraction 2 showing the retention time (red bar) in which the SDC1 peptide was isolated and extracted Ion Chromatogram (XIC) of the elution of the precursor (top). Detailed view of both TIC and XIC (bottom). C Fragmentation spectrum of the SDC1 precursor. D Normalized abundances (blue: grouped; gray: individual) of the SDC1 precursor in mimic and miR21− samples after processing with Proteome Discoverer 2.4 software. [file 13287_2023_3613_MOESM1_ESM.tif]

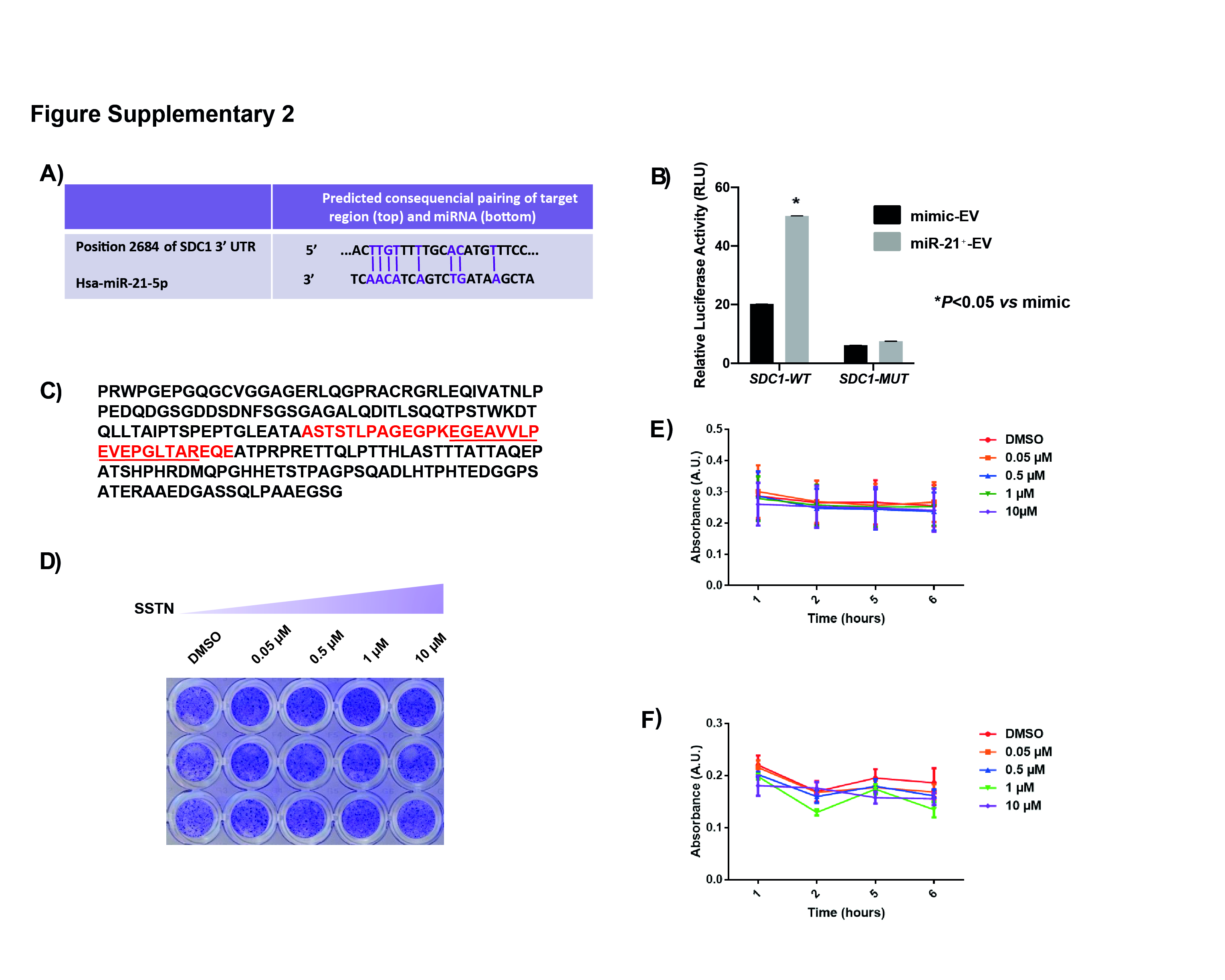

Supplement: Supplementary file 2 — Additional file 2: Fig. S2. SSTN Effectiveness. A Binding sites between SCD1 and miR-21 carried out using the http://www.targetscan.org/. B Relative luciferase activity was increased following treatment with a combination of mimic-EV or miR-21+-EV on MSC transfected with SDC1-WT or SDC1-MUT suggesting that miR-21 regulates SDC1. *P < 0.05 versus mimic treatment. The data are presented as the means ± EEM, analyzed by independent sample t test. The experiment was independently repeated 3 times. C SDC1 sequence by UniProt ID. In red the identified by our shotgun proteomic study and in underlined red SSTN sequence. D Representative plate of MSC stained crystal violet. E Crystal violet analysis of MSC treated with different amounts of SSTN (10 µM, 1 µM, 0.5 µM, 0.05 µM) to different times (1, 2, 5 and 6 h). F Cytotoxicity assays of MSC treated with different amounts of SSTN (10 µM, 1 µM, 0.5 µM, 0.05 µM) to different times (1, 2, 5 and 6 h). [file 13287_2023_3613_MOESM2_ESM.tif]

Figure 1F

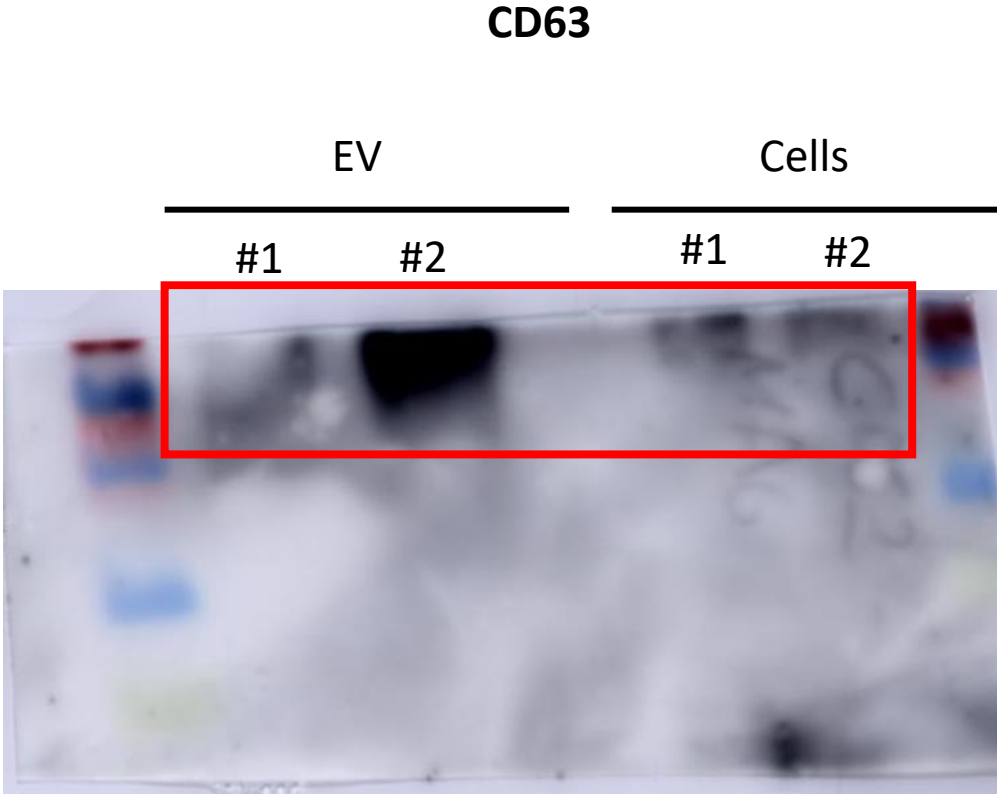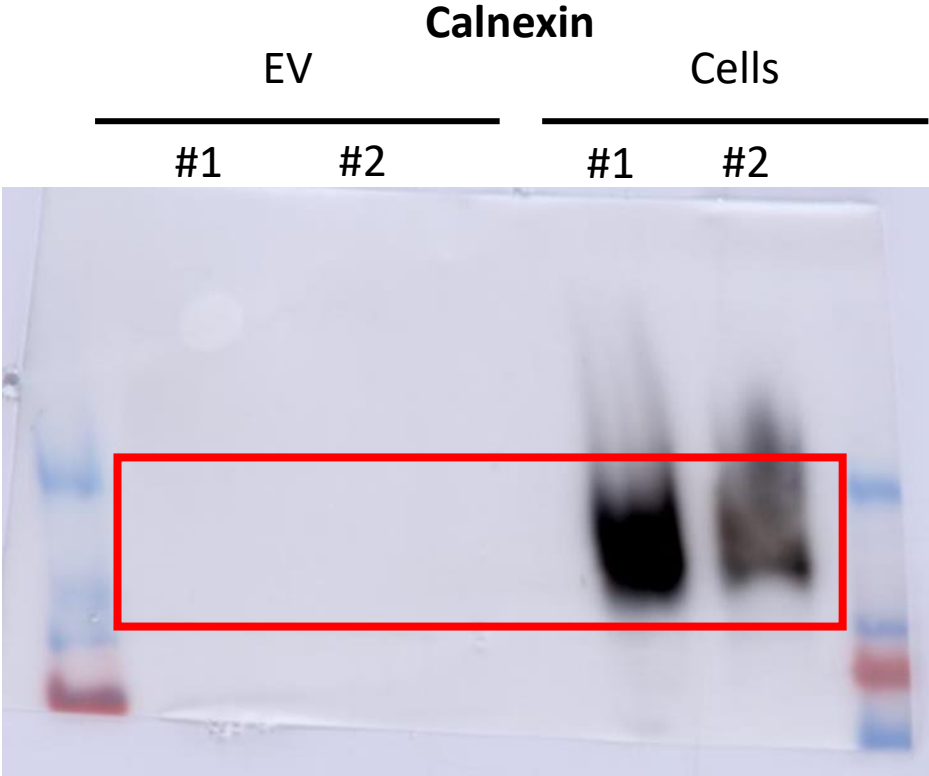

Figure 1F

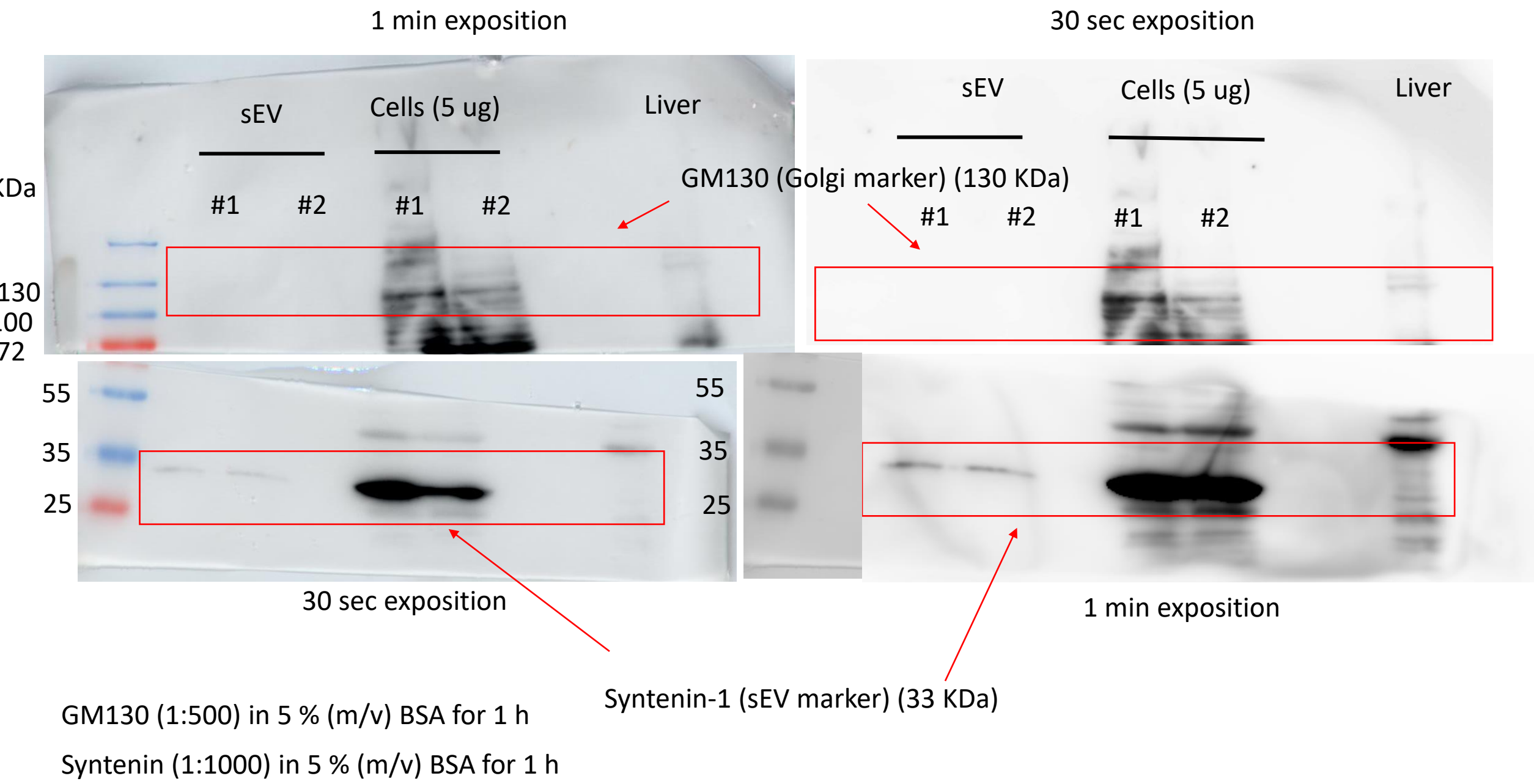

Figure 3C

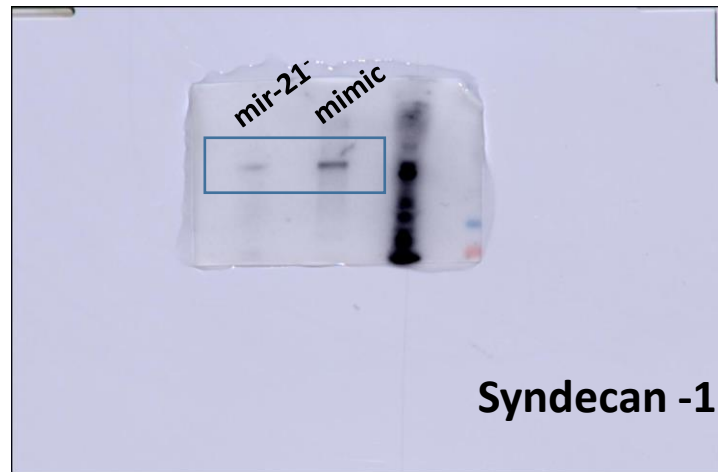

Syndecan -1

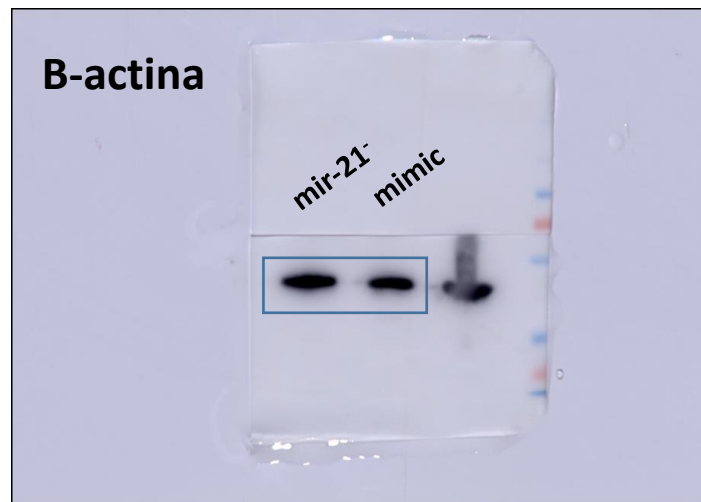

B-actina

Figure 4D

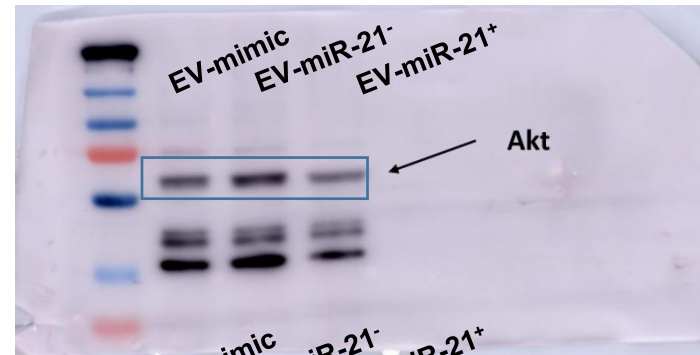

Akt

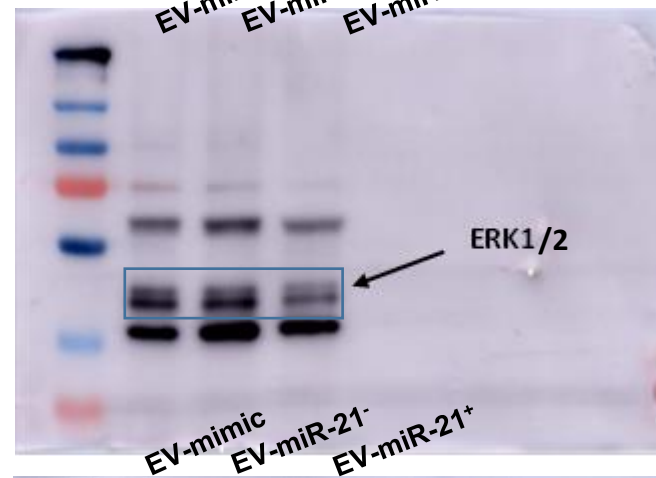

ERK1/2

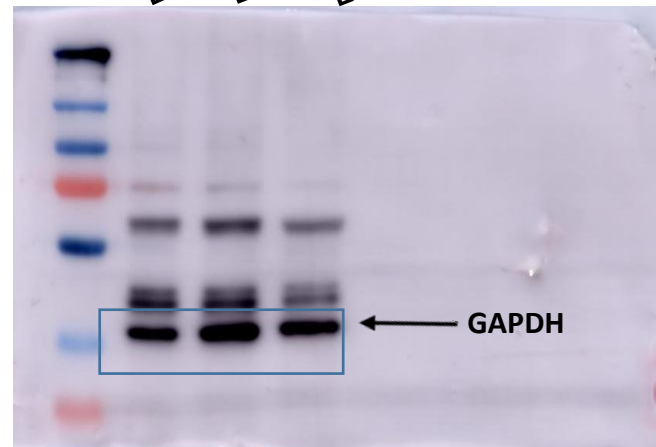

GAPDH

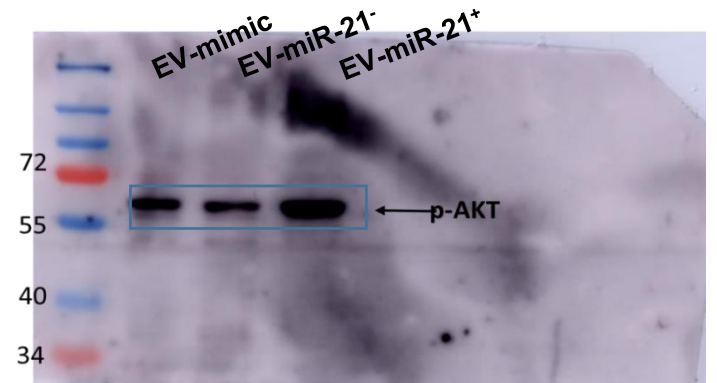

p-AKT

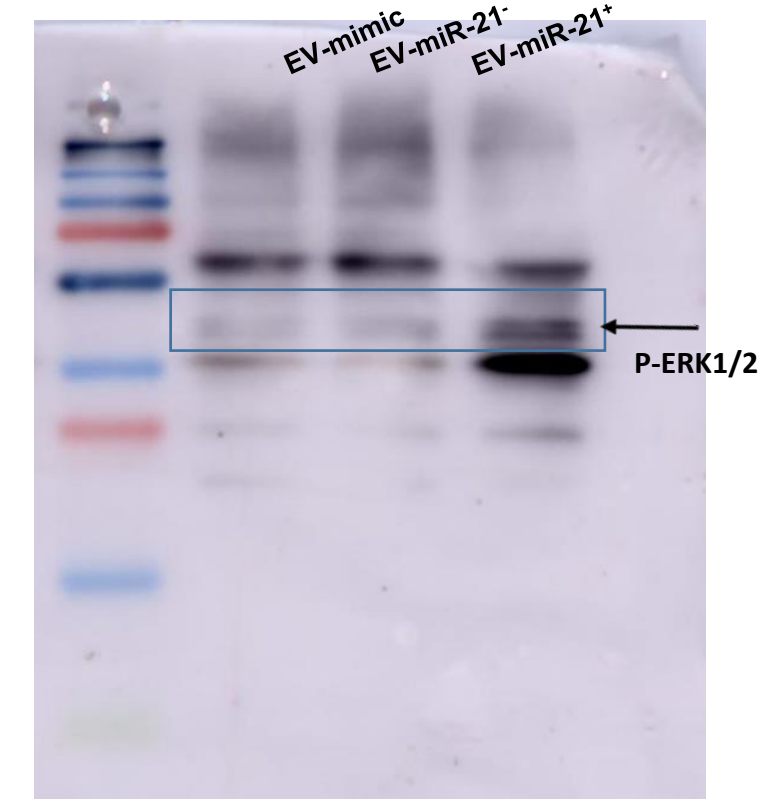

P-ERK1/2

Figure 4D

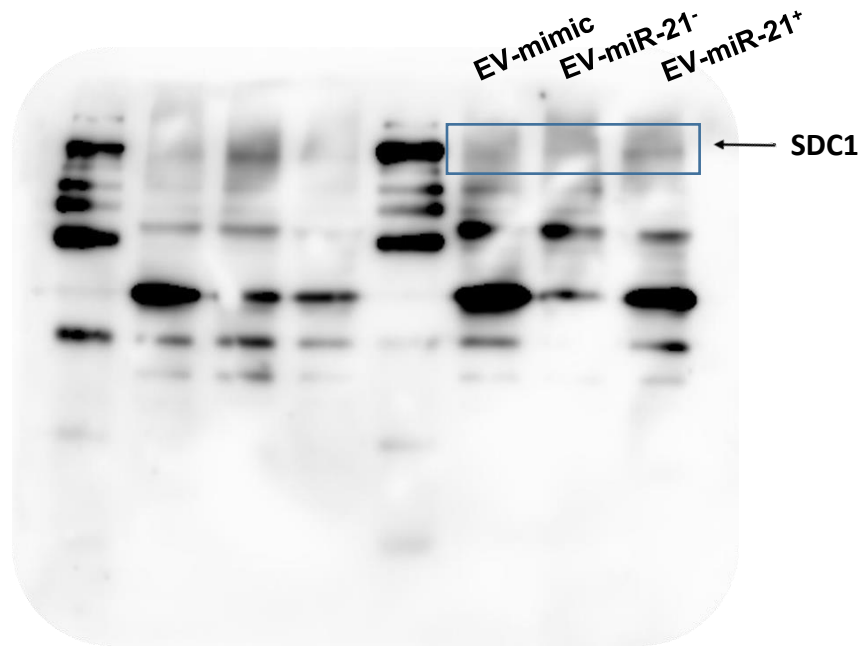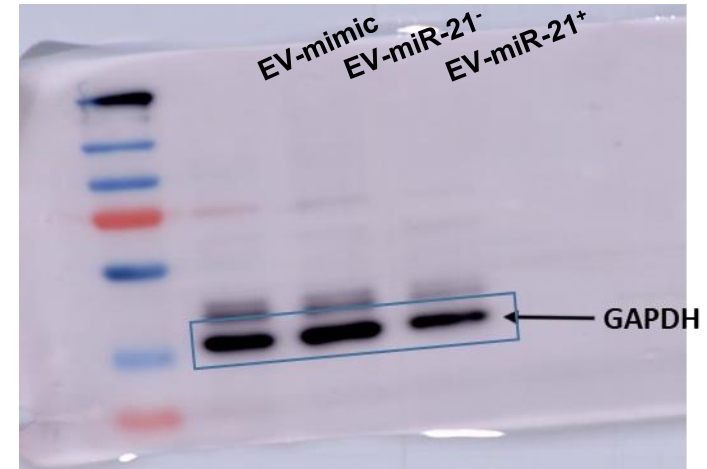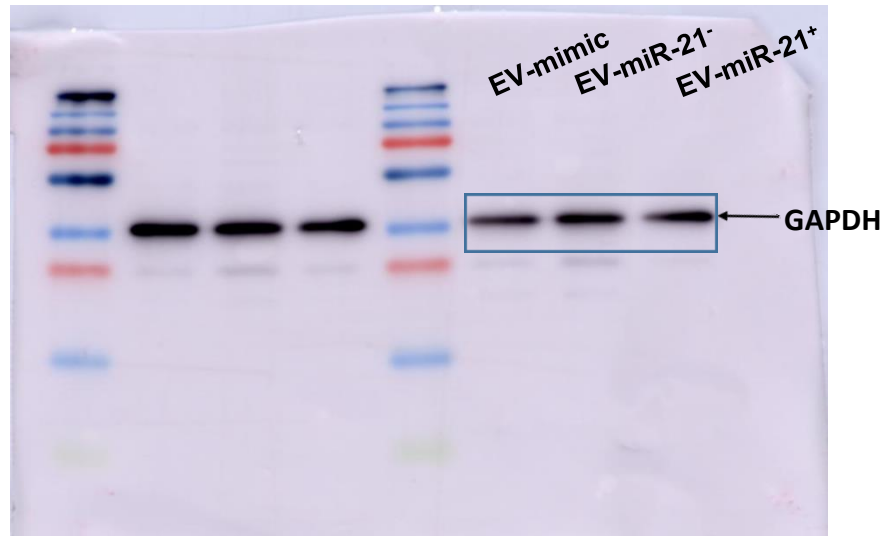

Supplement: Supplementary file 3 — Additional file 3: Fig. S3. Full-length blots/gels presented in the manuscript. [file 13287_2023_3613_MOESM3_ESM.pdf]
